# Lane departure on combined curves: driver heterogeneity, centrifugal risk, and crash prevention

**DOI:** 10.1038/s41598-026-37251-1

**Published:** 2026-02-12

**Authors:** Xiaomeng Wang, Yujie Zhang, Yi Li, Xiebowen Yi, Xuesong Wang, Guangjie Hao

**Affiliations:** 1 National Engineering Research Center of Road Safety Control Technology, No. 5, Boxing 2nd Road Beijing Economic-Technological Development Area, Beijing, 100176 China; 2 College of Transport & Communications, Shanghai Maritime University, No. 1550, Haigang Avenue, Shanghai, 201306 China; 3 Logistics Research Center, Shanghai Maritime University, No. 1550, Haigang Avenue, Shanghai, 201306 China; 4 College of Control Technology, Wuxi University of Technology, No. 1600 Gaolang West Road, Wuxi, 214121 Jiangsu China; 5 Key Laboratory of Road and Traffic Engineering of the Ministry of Education, Tongji University, No. 4800, Cao’an Road, Jiading District, Shanghai, 201804 China; 6 Qinghai Transportation Construction Management Co., Ltd., No. 26 Wenjing Street, Xining, 810023 Qinghai China

**Keywords:** Lane departure behaviour, Centrifugal force direction, Driver characteristics, Multivariate adaptive regression splines (MARS), Combined curves, Threshold value, Engineering, Mathematics and computing

## Abstract

Lane departure crashes were more likely to result in severe traffic crashes. This objective of this study was to explore the effects of drivers’ characteristics on the asymmetric lane departure behaviour in combined curves. A high degree of freedom driving simulator was used to collected vehicle operation data in a mountainous freeway. Totally 948 lane departure events were collected. The lane departure behaviour was classified into two categories based on centrifugal force direction: *In the Direction of Centrifugal Force* (IDCF) and *Against the Direction of Centrifugal Force* (ADCF). The average speed, maximum lateral departure and departure duration distance were analyzed. A Multivariate Adaptive Regression Splines (MARS) model was used to analyze nonlinear relationships between driver characteristics and maximum lane departure. It found that: (1) Sag-curves and crest-curves had higher average lane departure frequencies compared to downslope- and upslope-curves. (2) IDCF events showed greater departure (0.83 m > 0.41 m) and longer departure duration distance (70.07 m > 58.60 m) than ADCF events. (3) The years of driving, daily driving distance, driving experience, road expert type and departure duration distance had significantly influence on departure severity. Also, driver characteristics and lane departure duration distance had an interaction effect on departure severity. (4) Speed and road geometry design could jointly influence the degree of lane departure, e.g., speed significantly affects lane departure behaviour on downslope-curves and upslope-curves in the IDCF scenario. (5) The lane departure duration distance threshold (35.00–110.00 m), and speed threshold (86.77–108.63 km/h) were proposed for the lane departure process. The study provided a basis for the optimisation of ADAS systems, the development of targeted driver training for high-risk drivers, and the optimisation of visual guidance design for combined curves.

**Supplementary Information:**

The online version contains supplementary material available at 10.1038/s41598-026-37251-1.

## Introduction

 Lane departure accidents have become a key challenge for road safety worldwide due to their high fatality rate^1,2^. It referred that each year lane departures accident account for over half of all traffic fatalities in the United States^3^. In all crashes with passenger car occupant fatalities in Sweden in 2010, 46% were found to relate to lane departure without prior loss of control^4^. The main cause of lane departure accidents is failure to intervene in time at the initial stage of the departure, resulting in a collision with oncoming traffic or an obstacle after the vehicle has fully crossed the line. Understanding the factors influencing lane departure accidents is therefore a prerequisite for reducing such accidents.

Driver characteristics have a significant impact on lane departure behaviour. Existing studies have shown that: gender, more average kilometers driven in the last year had a significant effect on lane departure behaviour, males were found to be significant factors for having a higher proportion of poor lane departure performance, and drivers with more miles driven in the last year were found to have better lane departure performance^5^. Driving experience is also a significant variable that can modulate the number of lane departures, with experienced drivers departing the lane less frequently than novice drivers^6^.

Road combined curves can also affect driving behaviour. It found that the risk of accidents was higher on combined curves compared to single horizontal and vertical curves. The significant effects of geometric design features on lane departure varied by type of combined curve^7^. Chen et al. examined how the combined alignments affect the probability of lane excursion while controlling for other factors, they found that the main influencing factors are the horizontal curvature at the current segment, the difference in horizontal curvature within the 300 m adjacent upstream alignment, and downslope and upslope^8^.

Early prediction and precise control of lane departure through intelligent driving technology is a key breakthrough in reducing casualties^9^. Research has showed that driving risk prediction is important for the development of intelligent vehicles^10^. Collision risk modeling with multiple surrounding target vehicles is essential for host vehicle trajectory planning, especially considering challenging target vehicle lateral behaviors^11^. Liang et al. present an interaction-aware trajectory predictor based on transformer-transfer learning for safe motion planning in autonomous driving^12^. The predictor focuses on a vehicle-to-vehicle (V2V) interaction scenario involving an autonomous vehicle (AV) and a human-driven vehicle (HDV). ADAS such as LKA (Lane Keeping Assist) and LDW (Lane Departure Warning) can significantly reduce the risk of lane departure accidents, but their current ADAS system suffers from the following shortcomings: (1) weak adaptation to complex road geometries; and (2) insufficient consideration of driver characteristics; (3) insufficient consideration of the direction of lane departure. Previous studies have shown that the direction of centrifugal force could affect the lane departure behaviour^7^.

Understanding the influence of driver characteristics on driving behaviour can provide an important basis for personalised upgrading of ADAS^13^, and understanding the influence of road geometric design elements on driving behaviour can provide important information for optimising scenario adaptation of ADAS^14^. The objective of the present study is to analyse the lane departure behaviour of drivers in complex combined curve scenarios. To achieve this objective, the study is structured as follows:

Data from 36 drivers were collected using a high-freedom driving simulator on a mountainous freeway—focusing on vehicle speed and lane departure metrics under complex combined curve conditions. Lane departure behaviors were classified into two types based on curve direction: IDCF (In the Direction of Centrifugal Force) and ADCF (Against the Direction of Centrifugal Force). Scenario-specific analysis was conducted for four typical combined curves (downslope-curve, upslope-curve, sag-curve, and crest-curve). Quantitative models were developed using MARS to relate lane departure behavior to driver characteristics per curve type, leading to the proposal of two safety thresholds: lane *departure duration distance* and vehicle speed.

**Fig. 1:**
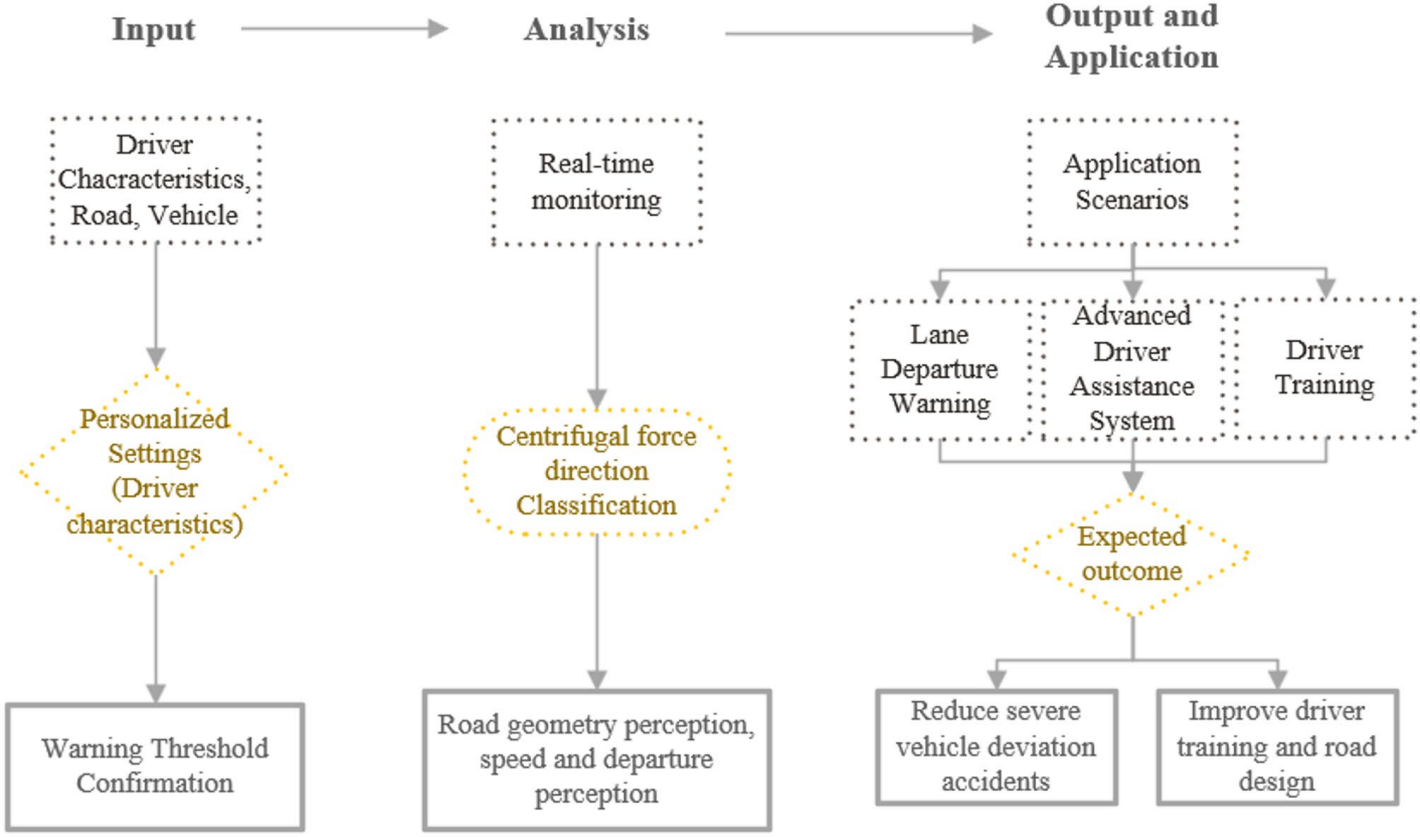
ADAS application flowchart.

The study provided a basis for the optimisation of ADAS systems, the development of targeted driver training for high-risk drivers, and the optimisation of visual guidance design for combined curves. We have included a flowchart illustrating how the findings of this study can be applied, as shown in Fig. 1 below.

## Literature review

### The impact of driver characteristics on driving safety

Researchers have shown that driver characteristics were important factors influencing driving skills, and that differences in driving skills could lead drivers to adopt different driving behaviours.

Research on elderly drivers (≥ 65 years old) indicates that declining cognitive perceptual assessment for driving are associated with increased driving accident risks. The deterioration of these cognitive dimensions may directly lead to inadequate lane-keeping ability, subsequently causing lane departures^15^. Seacrist et al. compared the rates of near-crashes, crash types, secondary tasks, and evasive manoeuvres among drivers of different ages. The results showed a significant decrease in the rate of near-crashes with increasing age^16^. Young drivers showed an overall increase in near-crash rates compared to adult and older drivers. Shaaban et al. provided insights into how driver characteristics influence driving safety under cognitive distraction^17^. The study showed that dangerous driving behavior had a direct effect on the crash risk probability.

Sawtelle et al. explored the relationship between driver characteristics and crash severity in rural lane departure crashes, emphasizing age, gender, and behaviour factors^18^. The analysis revealed that older drivers faced significantly higher risks of severe or fatal crashes compared to younger drivers. In contrast, male drivers exhibited lower crash severity odds than females across most scenarios. Risky behaviours such as speeding and driving under the influence amplified crash severity. Song et al. proposed a LC spatiotemporal feature model (LSFM) based on the driving behavior generation mechanism^19^. Hadadi et al. proposed non-lane-based discipline has an important role in changing car drivers’ driving behaviors from typical and sluggish to aggressive behavior compared with lane-based discipline^20^. The National Highways provides insights into how driver characteristics, such as age and behaviour influence road safety outcomes^21^. The report highlights that older drivers (aged 60 and above) are more likely to be involved in severe collisions. Furthermore, the report identifies key contributory factors to collisions, such as driver distraction.

### The influence of road geometry design on road safety

Complex combined curves could affect road traffic safety. Compared to other road geometries, mountainous freeways with a higher prevalence of horizontal and vertical curves were more likely to lead to lane departure events^7,22^.

Saleem & Persaud used a negative binomial model to develop a collision prediction model for curved sections and the preceding and following tangent sections of curves on rural two-lane freeways^23^. They examined the relationship between collisions under different traffic volumes and deflection angles. The results indicated that sections with steeper slopes had a higher crash reduction rate than those with flat slopes. Wang et al. used a Bayesian model to investigate the relationship between vehicle collision frequency on sag-curves and road geometric attributes, road configuration features, and traffic volume. The results showed that curve curvature, slope, and road speed limit influence crash frequency^24^. Wang et al. analyzed micro driving behaviour on four types of combined curves by using random forests and SHAP. The study highlighted the need to pay special attention to lane departure behaviour on upslope-curves and crest-curves, with the effect of road geometric design parameters varying between different combined curves^25^. Luo et al. presents new evidence of the effects of Transport infrastructure connectivity on conflict resolution by conducting a natural experiment and applying machine learning methods to overcome the endogeneity issue^26^.

Kazemzadehazad et al. used micro-driving behaviour as a measure of driver performance on combined curves and optimized the safety conditions of existing road curves by implementing warning signs^27^. Roadway geometric parameters significantly influenced lane departure risk by interacting with driver perception through mechanical mechanisms. Beyond the limitations of physical line-of-sight, combined alignment can also cause visual perception distortions for drivers. Analysis indicates that when horizontal curves overlap with crest longitudinal curves, drivers typically misjudge the horizontal curvature^28^. Xu et al. analyzed lateral acceleration experienced by drivers on mountainous highway alignments from a comfort perspective^29^. The study collected lateral acceleration distributions from drivers on 12 highways with varying design speeds and topographies, establishing a regression model for lateral acceleration versus curvature. It revealed a negative correlation between lateral acceleration and curvature, as well as a significant influence of design speed on lateral acceleration^30^.

Road geometry design also indirectly impacts safety by influencing drivers’ cognitive and behavioral decision-making processes. Complex alignments, particularly the combinations of consecutive curves and gradients common on mountain highways, significantly increase drivers’ mental workload by requiring simultaneous handling of lateral (steering) and longitudinal (braking) control tasks^31^. Research indicates that drivers tend to overestimate available safety margins on curves with insufficient sight distance, leading to excessive entry speeds that heighten risks of loss of control and lane departure^32^. Therefore, incorporating driver workload levels and sight distance conditions into safety assessments and design considerations is crucial for understanding accident causation and developing interventions—such as establishing appropriate recommended speeds. Research by Habib et al. highlights the importance of integrating human factors with physical design parameters by establishing curve-specific recommended speed limits based on the combination of psychological workload and available sight distance^33^. Insufficient sight distance exacerbates risks posed by sharp curves, steep gradients, or their adverse combinations, as it limits drivers’ ability to access critical information, thereby impacting their decisions regarding speed adjustment and trajectory control^34^.

### MARS model

In recent years, many researchers have used the MARS model to analyze traffic accident data. Compared with other models, the MARS model offers a balance between model interpretability and robustness in handling outliers, which improves prediction accuracy.

In the analysis of accident data, the MARS model showed good ability to handle outliers and achieved satisfactory prediction accuracy^35^. Murat analyzed outliers in the data set using the MARS model. The results indicated that MARS could capture clear observation sets when the outlier rate in the data was less than 30% and the sample size was not large^36^. Seefong et al. combined machine learning with MARS techniques to predict accident data, and the results showed that the MARS method could accurately predict accident data and determine factors that influence the severity of collision outcomes^37^.

In previous studies, several researchers have compared the MARS model with other regression and machine learning models in terms of their predictive performance. Haleem and colleagues analysed vehicle crash data over a four-year period from 2007 to 2010 and showed that the MARS model provided better fitting and prediction than the negative binomial model^38^. Li et al. compared the MARS model with models such as categorical regression trees, gradient boosted decision trees, random forests, and logistic regression. The results showed that although the complexity of the MARS model was much lower than that of the gradient boosted decision tree model, its prediction error rate was lower than that of other models except the gradient boosted decision tree model^39^.

In summary, the above studies provide a theoretical foundation for vehicle driver assistance systems and lane departure systems. Tawfeek & El-Basyouny stressing on the difference between driver behavior at intersections and on segments. Detecting intersection-related driving behavior, provides important theoretical foundations for the advancement of driver assistance systems^13^. Zhao et al. compared the driving performance of drivers with different ages after receiving lateral collision warnings. The results revealed that younger drivers responded to collision warnings more accurately than older drivers due to their stronger sensitivity^40^. Existing literature indicates that experience, gender and road environment affects driving performance^41–43^. Furthermore, since drivers with different characteristics perceive hazards differently, it is difficult to set an appropriate warning time^44^.

Based on the discussion above, the following points can summarize the research gaps that this paper will address:

1. Existing literature predominantly categorizes lane departure events based on macro-level classifications such as left/right or driving direction (straight/left/right turn). However, few studies have quantified and compared asymmetric departure behaviors using the direction of centrifugal force—a key dynamic characteristic during curved driving—as a classification criterion^45,46^.
  Actually, centrifugal forces significantly increase the probability of vehicles deviating from the lane during cornering compared to traveling tangentially. For instance, when horizontal curve radii are small and superelevation is insufficient, vehicles may not only drift toward the lane edge but also veer off the roadway entirely^47^.
2. Most early studies focused on relatively simple curve types, emphasizing single concave/convex curves or straight sections, with insufficient exploration of the systematic departure mechanisms under continuous combined alignments in mountainous highways^48,49^.
3. Previous studies on driver characteristics or road/speed factors have predominantly employed linear regression and similar methods for single-factor or stepwise control analysis, while there are few research on multi-factor interactions and nonlinear relationships^18,50,51^.
4. Although existing engineering standards and evaluation literature provide general recommendations and system performance metrics for LDW design, there is a lack of explicit speed or departure thresholds that can be directly applied to ADAS calibration under combined curve and driver stratification conditions^52,53^. On drivers’ behavior is essential for enhancing human-like automated driving from the perspectives of safety and comfort. And, it can provide a basis for improving the control strategies of Autonomous vehicles and optimizing advanced driver assistance systems^14,54^.

This paper aims to fill a gap in the field. At the same time, this paper innovatively classifies considering the direction of turning and the direction of centrifugal force, lane departure behavior is categorized into Against the Direction of Centrifugal Force (ADCF) and In the Direction of Centrifugal Force (IDCF). The average speed, maximum lateral departure and departure duration distance were analyzed. A Multivariate Adaptive Regression Splines (MARS) model was used to analyze nonlinear relationships between driver characteristics and lane departure.

## Data preparation

### Apparatus

**Fig. 2:**
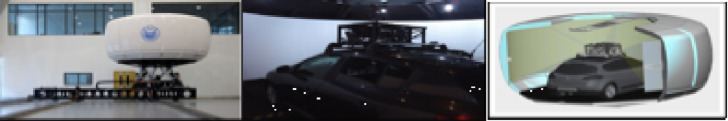
Tongji University driving simulator.

The equipment used in this experiment is the Tongji University driving simulator. The simulator dome contains a fully equipped Renault Megane III vehicle cab installed on an eight-degree-of-freedom motion system with an X-Y motion range of 20 × 5 m. The simulator used in this study is a motion-based simulator that provide immediate and realistic feedback to the driver. By replicating sensations such as driving over slopes, bumps, and super-elevations on test roads, the simulator creates an immersive environment for the user. In addition, the simulation system can provide drivers with rear view and side view images^47^. The validity of this driving simulator has been verified in previous studies^55^. The performance of the driving simulator was evaluated through three tests: simulator sickness, stopping distance, and traffic sign size recognition. The results demonstrated that the simulator met al.l validation criteria, with at least 75% of participants experiencing no simulator sickness, stopping the vehicle within 2 m of the stop line, and accurately judging the realism of the traffic sign size (Fig. 2).

In recent years, driving simulators have proved to be an effective tool for studying road safety and driving behaviour. Bella has conducted a series of studies on driving behaviour and road safety using the CRISS Driving Simulator^56^. Bella mentions that the simulator can effectively reproduce the effects of road geometric design on driving behaviour and comparing the simulated data with real-world road data, it was found that simulator data did not differ significantly from real-world data in most cases, especially in assessing driver reaction time, distance control, and driving speed. The results promoted the application of driving simulator technology in traffic safety research^57^. Studies by Alonso et al. and Aksan et al. further validated the effectiveness of driving simulators in road safety and driving behaviour research^58,59^.

Driving behavior in simulators may differ from real-world road driving behavior, but research indicates that their performance exhibits high similarity to actual road behavior. Simulators demonstrate good relative validity and serve as effective tools for studying road traffic safety^60^. In this study, the average speed of the combined curved road section was 94.55 km/h, and the speed of the real road is expected to be lower than the simulated road speed. The validity of the past data of the driving simulator used in this study were tested, and the t-test results showed that the difference between the simulated data and the real data was not significant^47^.

### Experimental design

#### Test road

The experiment was based on a four-lane freeway in Hunan Province, China. A 24-kilometer test road was constructed using 3D Studio MAX, with a designed speed limit of 100 km per hour, the horizontal curve radii are all greater than 1000 feet (305 m). Seventy-one combined curves were selected included 24 downslope-curves, 21 upslope-curves, 12 sag-curves, and 14 crest-curves.

**Fig. 3:**
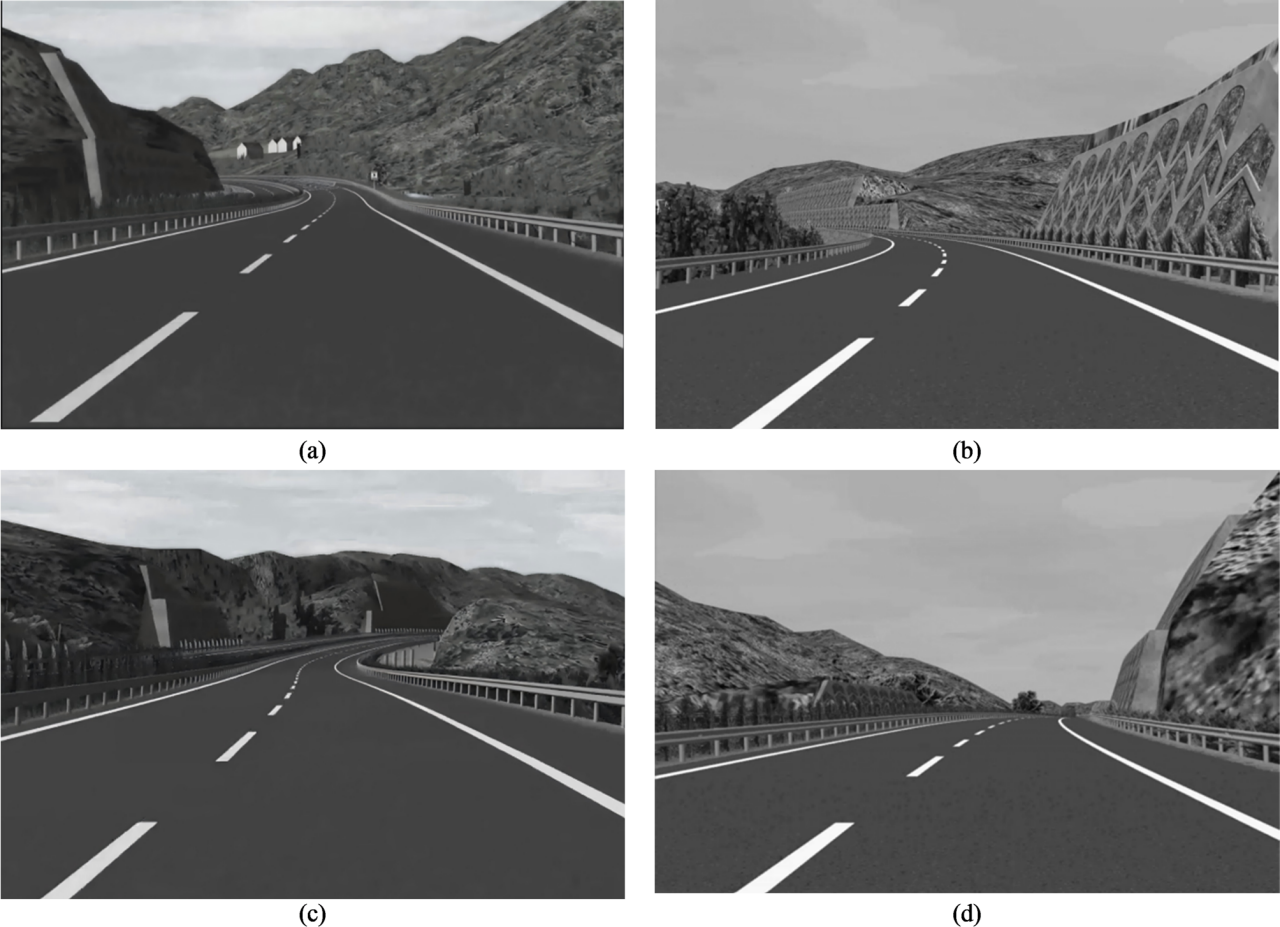
Configuration of the four types of combined curves. (**a**) Downslope-curve, (Vertical curve of road section is a downslope, combined with a horizontal curve), (**b**) Upslope-curve, (Vertical curve of road section is an upslope, combined with a horizontal curve), (**c**) Sag-curve, (Vertical curves of road section is a sag-curve, combined with a horizontal curve), (**d**) Crest-curve, (Vertical curves of road section is a crest-curve, combined with a horizontal curve)

The illustrations of the four types of combined curves are shown in Fig. 3.

The radius, slope, and other data characteristics of the four types of combined curves are shown in Table 1.

**Table 1:** Geometric design characteristics of combined curves.

Curve type	Explanation	Mean	Standard	Min	Max
Downslope-curve	Mean slope (%)	2.49	1.38	0.76	5.51
	Slope differential of maximum and minimum slope (%)	2.01	1.5	0	4.58
	Length (m)	404.4	149.24	222.51	790.76
	Length of circular curve (m)	197.01	130.9	42.51	490.76
	Length of approach transition (m)	103.48	21.15	80	150
	Length of departure transition (m)	103.91	19.85	80	150
	Radius (m)	862.66	381.23	400	1648.13
	Superelevation (%)	0.5	0.3	0.3	0.7
Upslope-curve	Mean slope (%)	2.49	1.51	0.09	5.25
	Slope differential of maximum and minimum slope (%)	2.27	1.7	0	5.9
	Length (m)	405.95	133.54	225	675
	Length of circular curve (m)	204.05	126.48	35	420
	Length of approach transition (m)	102.14	27.907	10	160
	Length of departure transition (m)	99.76	28.327	0	155
	Radius (m)	917.11	509.68	400	2500
	Superelevation (%)	0.5	0.3	0.3	0.7
Sag-curve	Mean slope (%)	– 0.4342	1.1503	– 2	1
	Slope differential of maximum and minimum slope (%)	4.8747	1.7514	0.73	8.1
	Length (m)	448.33	97.654	285	595
	Length of circular curve (m)	238.75	87.517	100	355
	Length of approach transition (m)	102.5	19.659	80	145
	Length of departure transition (m)	107.08	19.655	85	140
	Radius (m)	543.83	138.82	400.25	720
	Superelevation (%)	0.5	0.3	0.3	0.7
Crest-curve	Mean slope (%)	– 0.1621	1.0721	– 2.3	1.18
	Slope differential of maximum and minimum slope (%)	3.45	1.4655	0.7	5.68
	Length (m)	407.36	140.75	240	585
	Length of circular curve (m)	215.93	126.8	65	385
	Length of approach transition (m)	94.64	31	0	140
	Length of departure transition (m)	96.79	30.78	0	135
	Radius (m)	856.854	534.634	487.16	2500
	Superelevation (%)	0.5	0.3	0.3	0.7

#### Driver characteristics

Prior to the experiment, all participants were provided with a written consent form that explicitly included:

- The purpose of the study and expected duration,
- Detailed description of experimental tasks,
- Types of data to be collected,
- Data usage plans,
- Contact information of the principal investigator for queries.

This study was approved by the ethics committee of Tongji Hospital (Approval Date: 11 November 2010). All methods were performed in accordance with the Declaration of Helsinki and relevant institutional regulations.

There are 36 participants in the driving simulator experiment, with 32 males and 4 females. Thirty-six can be used as an acceptable sample size in road safety assessments on mountainous freeways because the road conditions and metrics used in driving simulation experiments are same^61–64^. The participants included various professions, such as designers, teachers, students, and drivers. Notably, 69.44% of the participants had experience driving on mountainous freeways, and 80% fell within the age range of 25 to 50 years. To facilitate subsequent model construction, driver characteristics were categorized into multiple classes. The criteria for dividing the intervals of age and years of driving were based on the recommendations from Song et al.^65^. Partial relevant statistical data for driver characteristics are presented in Table 2.

**Table 2:** Descriptive statistics for categorical driver characteristics.

Attribute	Code name	Category	Frequencies (pcs)		Percentage (%)
Gender	CV1	Female	0	4	11.12
		Male	1	32	88.88
Age (years)	CV2	20–24	1	3	8.33
		25–34	2	13	36.11
		35–49	3	16	44.44
		50–64	4	4	11.11
Years of driving experience (year)	CV3	0–4	1	12	33.33
		5–9	2	11	30.55
		10–19	3	8	22.22
		20–34	4	4	11.11
		35–49	5	1	2.77
Freeway driving frequency	CV4	Never	1	3	8.33
		occasionally	2	12	33.33
		Frequently	3	19	52.77
		Daily driver	4	2	5.55
Driving experience on mountainous freeways	CV5	Yes	1	25	69.44
		No	2	11	30.56
Road Expert	CV6	No	1	21	58.33
		Road traffic planning designers	2	11	30.55
		Road traffic safety technicians	3	4	11.11
Driving license type	CV7	Large passenger vehicles	1	3	8.33
		Medium and heavy full and semi-trailer trucks	2	1	2.77
		Medium passenger vehicle	3	2	5.55
		Medium, heavy goods vehicles	4	3	8.33
		Small passenger vehicle	5	21	58.33
		Small automatic cars	6	8	16.69
Average daily driving time on workdays (h)	CV8	< 0.5	1	5	13.88
		0.6–1.0	2	9	25.0
		1.1–1.5	3	2	5.55
		1.6–2.0	4	2	5.55
		2.1–2.5	5	2	5.55
		2.6–3.0	6	4	11.11
		3.1–3.5	7	2	5.55
		3.6–4.0	8	2	5.55
		> 4.0	9	8	22.26
Average daily driving distance on workdays (km)	CV9	< 5	1	6	16.67
		6–10	2	3	8.33
		11–15	3	0	0.00
		16–20	4	2	5.55
		21–30	5	3	8.33
		31–40	6	2	5.55
		41–60	7	6	16.67
		61–80	8	4	11.11
		> 80	9	10	27.79
Average kilometers driven in the past year (km)	CV10	< 5000	1	8	22.22
		5001–10,000	2	7	19.44
		10,001–15,000	3	4	11.11
		15,001–20,000	4	4	11.11
		20,001–25,000	5	1	2.77
		> 25,000	6	12	33.35
Mountainous freeway driving frequency	CV11	Never	1	13	36.11
		Occasionally	2	20	55.56
		Frequently	3	3	8.33
Experience of minor traffic accidents	CV12	Yes	1	25	69.45
		No	0	11	30.55
Driving experience on driving simulator	CV13	Yes	1	21	58.34
		No	2	15	41.66
Driving frequency	CV14	Never	1	2	11.11
		Occasionally	2	8	16.67
		Frequently	3	26	72.22
Types of vehicles usually driven	CV15	Automatic transmission	1	9	25
		Manual transmission	2	27	75

Among the drivers participating in the experiment, 88.88% are male and 11.12% are female. Over 90% of the drivers are over 25 years old. Additionally, 60% of them have more than 4 years of driving experience.

#### Experiment procedure

**Fig. 4:**
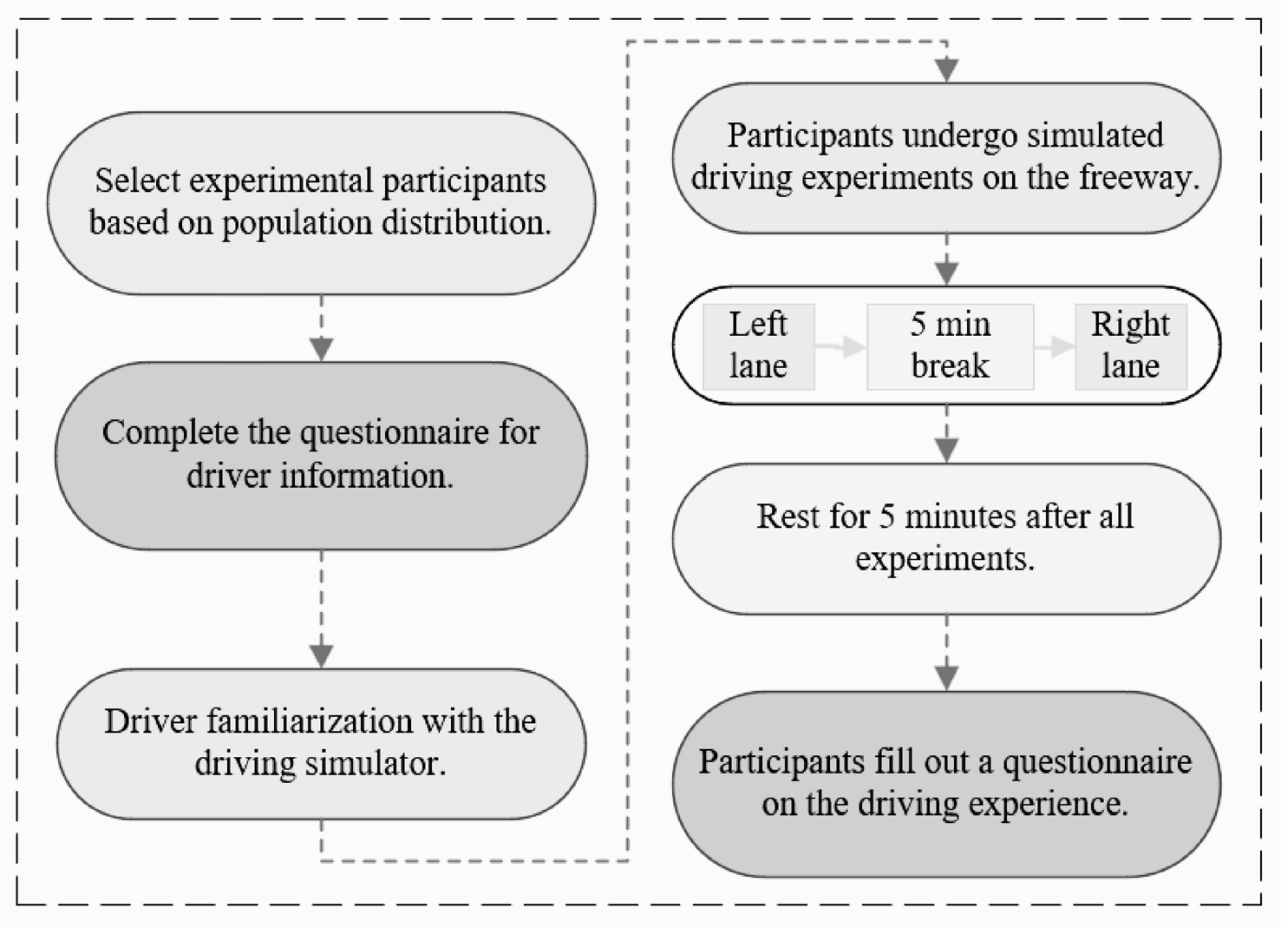
Driving simulator experiment flowchart.

The driving scenario in this experiment is assumed to be in free-flow traffic. Each driver conducts a simulated drive on both the left and right directions, with a five-minute rest after each direction drive. Drivers need to keep driving in their original lane during the experiment and not change lanes at will. And drive according to the speed limit. The specific experimental procedure is as shown in Fig. 4.

The lane departure data that resulted from the drivers’ operation of the vehicle was measured and recorded at a frequency of 20 Hz. These data were then averaged over five-meter segments and were related to the roadway markers.

## Lane departure analysis

### Lane departure classification

**Fig. 5:**
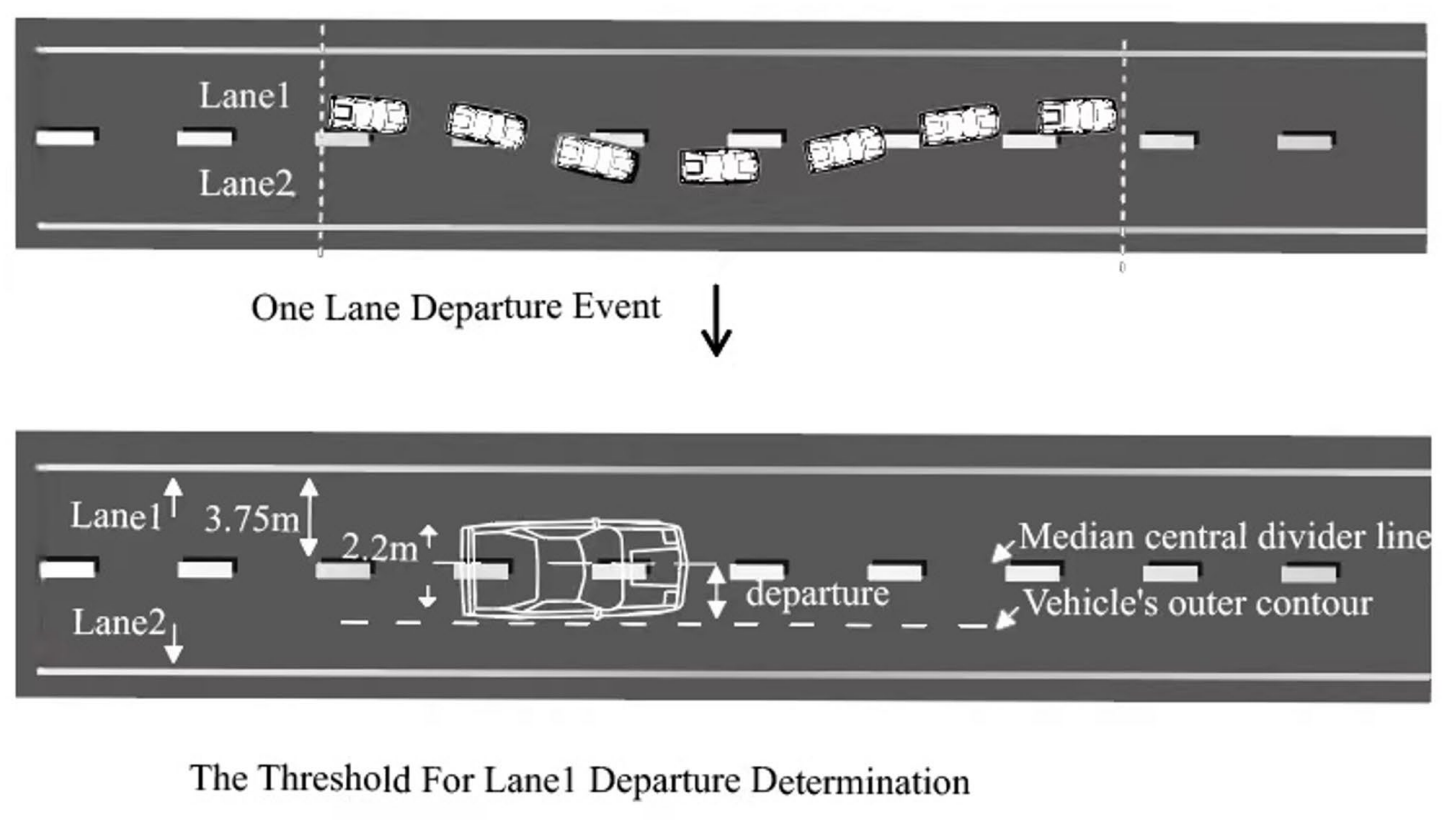
Schematic diagram of a lane departure scenario.

In this study, the lane departure event is defined as the moment when the vehicle’s outer contour crosses the lane boundary line. The width of the vehicle used in the driving simulator is 220 cm and the lane width is 375 cm. The threshold value for lane departure is therefore 77.5 cm, which is shown in Fig. 5.

The influence of the vehicle deviates to the inside or the outside of the lane in a curve section are different^25^. The recorded steering data serve as the basis for distinguishing the relationship between the departure of the vehicle and the direction of the centrifugal force. In the event of the vehicle trajectory being deflected towards the interior of the curve, this is known as the In the Direction of Centrifugal Force (IDCF) departure event. Conversely, when the vehicle trajectory is deflected towards the exterior of the curve, this is known as the Against the Direction of Centrifugal Force (ADCF) departure event.

**Fig. 6:**
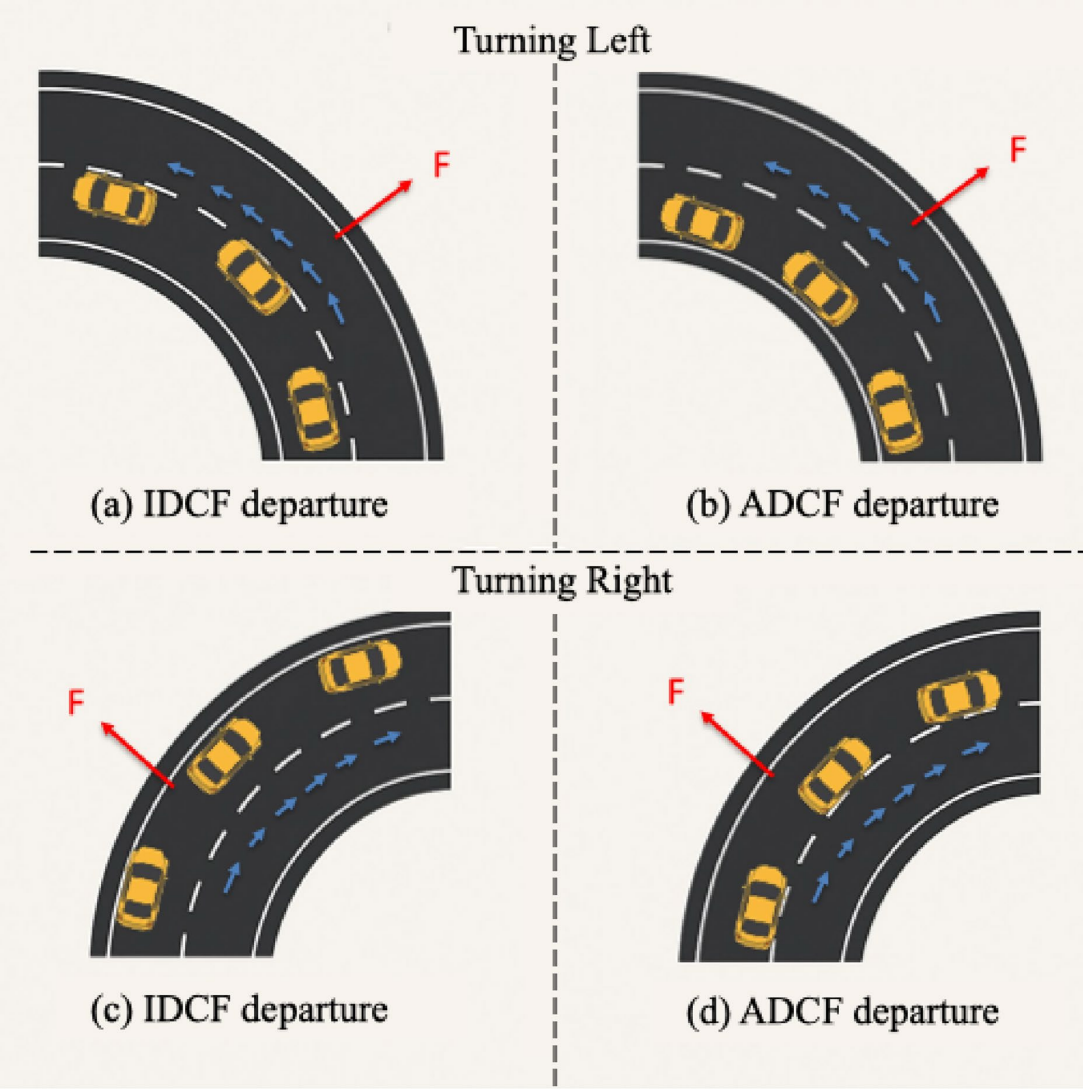
ADCF and IDCF categorization diagrams.

The specific classification is illustrated in Fig. 6. When a vehicle deviates from the centre line of the lane during a left turn and veers to the outside of the bend, it is categorised as an IDCF departure event, as demonstrated in Fig. 6 (a). Conversely, when a vehicle deviates to the inside of the curve, it is designated as an ADCF departure event, as shown in Fig. 6 (b). It is important to note that this classification system is equally applicable to right-hand turns, as illustrated in Fig. 6 (c) and (d). The initial departure value is defined as the vertical distance between the vehicle centreline and the road median.

### Occurrence frequency comparison of combined curves

**Fig. 7:**
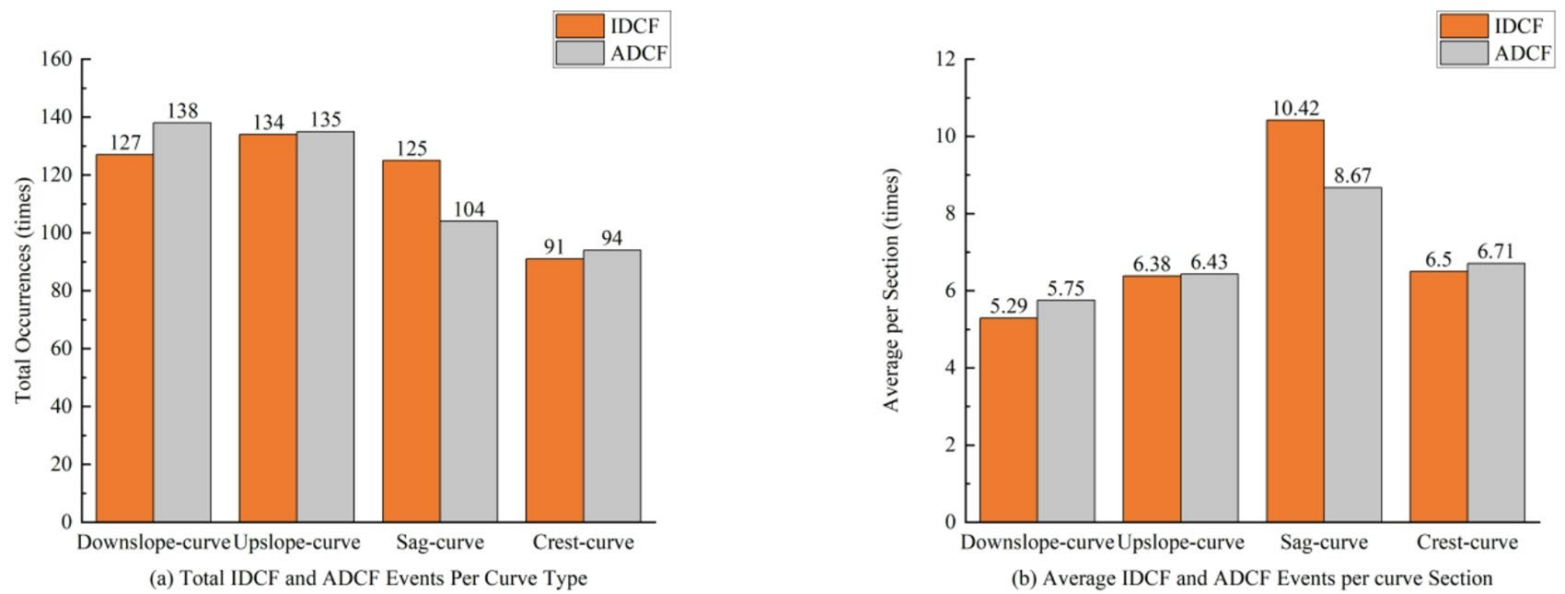
ADCF and IDCF occurrence frequency comparison of combined curves.

For the lane departure event data, data from the initial vehicle start and the near-end portion part with high variability were excluded. Finally, 948 lane departure events were identified from the driving simulator experiment. The total IDCF and ADCF events per curve type and average IDCF and ADCF events per curve section are shown in Fig. 7.

From Fig. 7, the four types of combined curves can be compared, the average occurs count of lane departure per section, and show that the average occurs count of downslope-curve and upslope-curve and crest-curve sections does not differ much, while the average occurs count in the sag-curves is larger. Comparison of the lane departure behaviour between the two types of IDCF and ADCF reveals that the frequency of ADCF is greater than that of IDCF on downslope-curve, upslope-curve, and crest-curves. However, the frequency of IDCF is greater than that of ADCF on sa*g*-curve only.

### Maximum lateral departure and departure duration distance

*Maximum lateral departure* (MLD) is defined as the maximum lateral distance between the vehicle centerline and the centerline of the carriageway and can represent the degree of lane departure. The *departure duration distance* (DPD) is the distance that a lane departure event lasts, from the start of the lane departure to the end of the lane departure.

**Fig. 8:**
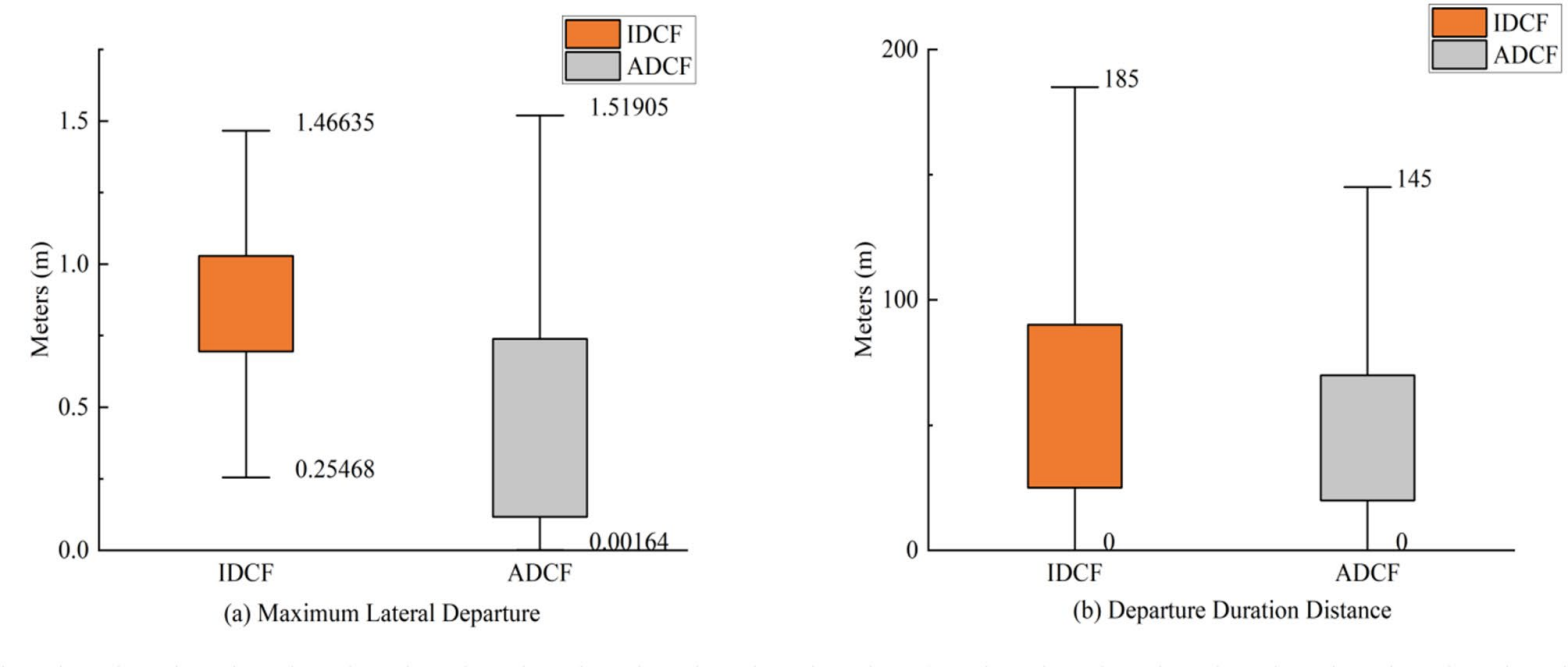
The descriptive statistic for different centrifugal force departure scenarios.

The *maximum lateral departure* (MLD) and *departure duration distance* (DPD) are analyzed. The descriptive statistical results for different centrifugal force departure scenarios are presented in Fig. 8.

Figure 8 shows that the *maximum lateral departure* (MLD) (1.52 m) for ADCF departure events exceeds the *maximum lateral departure* (MLD) for IDCF departure events (1.47 m). While ADCF departure events may produce larger departure under extreme conditions, its lower bound of performance range is also lower. In contrast, the *maximum lateral departure* (MLD) for IDCF departure events (ranging from 0.25 m to 1.47 m) are more concentrated, exhibiting greater stability and reduced variability.

When comparing the duration distances of the lane departure events, we can see that the longest *departure duration distance* (DPD) of the IDCF departure event is 185 m, which is greater than the 145 m of the ADCF departure event. The lane departure events consistent with centrifugal force have a longer *departure duration distance* (DPD) and the driving behaviour against centrifugal force has a shorter *departure duration distance* (DPD).

Although the descriptive statistics above indicate that the *departure duration distance* (DPD) for IDCF is longer than that for ADCF, this difference may stem from random variation. To assess whether this discrepancy is statistically significant, we conducted a normality test on the *departure duration distance* (DPD) of the IDCF and ADCF events. The results indicate that the data does not follow a normal distribution. Nonparametric tests perform well when examining non-normally distributed data. Given that the two data sets exhibit similar levels of dispersion, they satisfy the “homogeneity of variance” assumption required for the Mann-Whitney U test, rendering the test results reliable. Therefore, we will consider the Mann-Whitney U test. The test results are shown in the table below (Table 3):

**Table 3:** Departure duration distance Mann-Whitney U test results.

Mann-Whitney U test	
Wilcoxon W	174,313
Test statistic	81,217
Standard error	3606.188
Standardized test statistic	− 2.816
Progressive significance (two-tailed test)	0.005

A Mann-Whitney U test was conducted to compare IDCF *departure duration distance* (DPD) with ADCF *departure duration distance* (DPD), examining whether these two types of lane *departure duration distance* (DPD) exhibit significant differences. Let $\:{\tau\:}_{IDCF}$ represent the mean of IDCF *departure duration distance* (DPD) and $\:{\tau\:}_{ADCF}$ represent the mean of ADCF *departure duration distance* (DPD). The following hypotheses are first established:

- Null hypothesis: $\:{\tau\:}_{ADCF}-{\tau\:}_{IDCF}=$0. The overall distribution of the two sets of data is identical (i.e., there is no difference in the *departure duration distance* (DPD) between the in the direction of centrifugal force and against the direction of centrifugal force).
- Alternative hypothesis: $\:{\tau\:}_{ADCF}-{\tau\:}_{IDCF}\ne\:$0. There is a significant difference in the overall distribution between the two sets of data (i.e., the *departure duration distance* (DPD) between the in the direction of centrifugal force group and against the direction of centrifugal force group is different). When *p* < 0.05, reject the null hypothesis and accept the alternative hypothesis.; when *p* > 0.05, accept the null hypothesis.

The results of the Mann-Whitney U test analysis show: *p* < 0.05. The null hypothesis is rejected, and the alternative hypothesis is accepted, indicating a significant difference between IDCF *departure duration distance* (DPD) and ADCF *departure duration distance* (DPD). Specifically, the *departure duration distance* (DPD) of IDCF events is significantly greater than that of ADCF *departure duration distance* (DPD).

Similarly, we conducted a normality test on the lane departure events of the IDCF and ADCF events. The results indicate that the data does not follow a normal distribution. Nonparametric tests perform well when examining non-normally distributed data. Given that the two data sets exhibit similar levels of dispersion, they satisfy the “homogeneity of variance” assumption required for the Mann-Whitney U test, rendering the test results reliable. Therefore, we will consider the Mann-Whitney U test, The test results are shown in the table below (Table 4):

**Table 4:** Departure events Mann-Whitney U test results.

Mann-Whitney U test	
Wilcoxon W	127,153.5
Test statistic	34,057.5
Standard error	3610.503
Standardized test statistic	− 15.874
Progressive significance (two-tailed test)	$\:<\hspace{0.17em}$0.001

Let $\:{\mu\:}_{IDCF}$ represent the mean of IDCF departure and $\:{\mu\:}_{ADCF}$ represent the mean of ADCF departure. The following hypotheses are first established:

- Null hypothesis: $\:{\mu\:}_{ADCF}-{\mu\:}_{IDCF}=$0. The overall distribution of the two sets of data is identical (i.e., there is no difference in the departure events between the in the direction of centrifugal force and against the direction of centrifugal force).
- Alternative hypothesis: $\:{\mu\:}_{ADCF}-{\mu\:}_{IDCF}\ne\:$0. There is a significant difference in the overall distribution between the two sets of data (i.e., the departure events between the in the direction of centrifugal force group and against the direction of centrifugal force group is different). When *p* < 0.05, reject the null hypothesis and accept the alternative hypothesis.; when *p* > 0.05, accept the null hypothesis.

The results of the Mann-Whitney U test analysis show: *p* < 0.05. The null hypothesis is rejected, and the alternative hypothesis is accepted, indicating a significant difference between IDCF departure value and ADCF departure value. Specifically, the departure value of IDCF events is significantly greater than that of ADCF departure value.

This indicates that when a driver is driving on a curve, the degree of lane departure in the same direction as the centrifugal force is a little more severe, but the lane departure values are more stable. During driving, the vehicle may make the driver feel safer when travelling along the inside of the lane, but when the driver is fighting against the centrifugal force, the degree of lane departure is less severe and the resulting departure values fluctuate to some extent and the duration distance is shorter.

### Average speed of lane departure behaviour

**Fig. 9:**
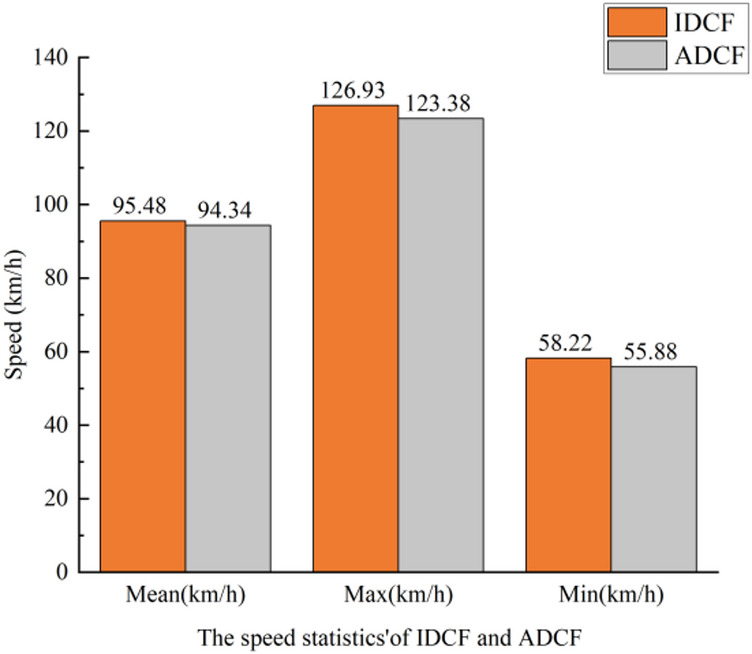
The speed statistics of IDCF and ADCF.

The average speed for IDCF event is 95.48 km/h, and the average speed for ADCF event is 94.34 km/h. Overall, there is little difference in speeds between the two types of lane departure behaviour. As shown in Fig. 9.

In summary, the driving behavior characteristics of ADCF and IDCF are summarized in the table below, which includes the mean and standard deviation for each variable (Table 5).

**Table 5:** The driving behavior characteristics for ADCF and IDCF.

Variable	Centrifugal force direction	Mean	S. D
Speed	IDCF	95.48 km/h	11.27
	ADCF	94.34 km/h	10.84
Departure duration distance	IDCF	70.07 m	68.14
	ADCF	58.60 m	60.12
Maximum lateral departure	IDCF	0.83 m	0.29
	ADCF	0.41 m	0.38

## MARS modeling

### MARS model

The MARS model was used in the research of this study. Friedman defines the MARS model as a multivariate, piecewise regression technique that can be used to model complex relationships^66^. MARS divides the predictive variable space into multiple nodes and fits a spline function between these nodes. The elements used to fit the MARS model are referred to as basic functions, and each basis function can represent either the main effects or interactions between variables. A generic MARS model is defined as follows:

1 $$\:\widehat{y}={\alpha\:}_{0}+\sum\:_{f}^{F}{\alpha\:}_{f}{\beta\:}_{f}\left(x\right)$$

In which, $\:\widehat{y}$ represents the predicted response variable, $\:{\alpha\:}_{0}$ is the coefficient of the constant basis function, $\:{\alpha\:}_{f}\:$ is the coefficient of the $\:f$-th basis function, $\:{\beta\:}_{f}\left(x\right)$ is the $\:f$-th basis function, and $\:F\:$is the total number of basis functions in the model.

Unlike other well-known parametric linear regression techniques, MARS offers a high degree of flexibility in studying the nonlinear relationships between input and response variables^67^. Moreover, the MARS model excels at revealing underlying connections in high-dimensional datasets and capturing apparent complex structures within data points^68^. The main research objective of this paper is to investigate the primary factors influencing lane departure.

This study focuses more on exploring the interrelationships between multiple variables. The MARS model can effectively handle data fitting with multiple independent variables. Therefore, the MARS model is a suitable fitting method for this study.

In the first step, known as the *construction phase*, a forward stepwise approach was used to add basis functions related to *departure duration distance* (DPD) and *maximum lateral departure* (MLD) to the model. The selection of predictive factors and node positions that had a significant contribution to *departure duration distance* (DPD) and *maximum lateral departure* (MLD) was made. During this phase, interactions between various driver characteristics were also introduced to examine if they could enhance the fit of the model.

In the second step, referred to as the *pruning phase*, a backward elimination process was used to remove the least contributing basis functions. To address overfitting, the article employed a generalized cross-validation statistic to penalize model complexity.

The final step was the *selection phase*, where the best MARS model was chosen from a set of recommended models based on the fit of the model and predictive ability.

The *Mean Absolute Deviation* (*MAD*) and *Mean Squared Error* (*MSE*) are commonly used to evaluate the predictive accuracy of the model^69^. In this study, 20% of the total data set was randomly selected as the test set to evaluate the predictive accuracy of the model^70^. The *MAD* and *MSE* can be calculated using the following formulas:

2 $$\:MAD=\frac{1}{n}\sum\:\left|{y}_{i}-{\mu\:}_{i}\right|$$

3 $$\:MSE=\frac{1}{n}\sum\:{({y}_{i}-{\mu\:}_{i})}^{2}$$

Where $\:n$ is the sample size of the prediction dataset, $\:{y}_{i}$ represents the departure value of event $\:i$, $\:{\mu\:}_{i}$ is the predicted departure value for event $\:i$. Due to its high sensitivity to data variations, MSE is chosen as one of the evaluation metrics of model accuracy in this study.

### Model establishment

The MARS model can be adjusted by modifying model parameters to change the number of terms and the maximum degree of interaction terms in the fitted model. While appropriate interaction terms can improve model accuracy, overly complex interactions can adversely affect model interpretability. Following the recommendations of Park et al.^71^, this study limits the degree of MARS basis functions to a range of 2–3 to avoid overly complex and difficult-to-interpret interaction terms.

#### Data standardization

In addition, due to the large differences in scale among continuous variables, scale was used to normalize continuous variables in the study, resulting in data standardized to a distribution with a mean of zero and a standard departure of one.

To eliminate the impact of dimensional differences between features on model training, this study performed standardized preprocessing on continuous variables of each model, this process transforms each feature into a standard normal distribution with a mean of 0 and a standard deviation of 1. Thereby eliminating the impact of differing feature dimensions on each model. For a sample value x in the feature matrix, the normalized value is calculated as shown in the following formula:

4 $$\:Z=\frac{X-\mu\:}{\sigma\:}$$

Among these: X: original value, $\:\mu\:$: the mean of the feature, $\:\sigma\:$: standard deviation.

The following model constructions were performed based on this standardized data.

#### Modeling approach

Finally, 948 lane departure events identified from the driving simulator were used to construct the MARS model. The classified driver characteristic data and combined curves were imported into R, and the earth_5.3.2 package was used to analyze the relationships between *maximum lateral departure* (MLD), *departure duration distance* (DPD), and various driver characteristic variables under IDCF and ADCF departure event of the four combined curves. This relationship is represented by constants, main effect functions, and interaction term functions, along with their corresponding coefficients.

After the lane departure data were classified and imported into the model, the data were split into a training dataset (80%) and a test dataset (20%) according to the specified ratio. Variables were categorized as either a main effect function or a function depending on whether they interacted with other variables. Interaction term functions could only include two to three variables due to the limitations of the maximum degree of base functions.

**Fig. 10:**
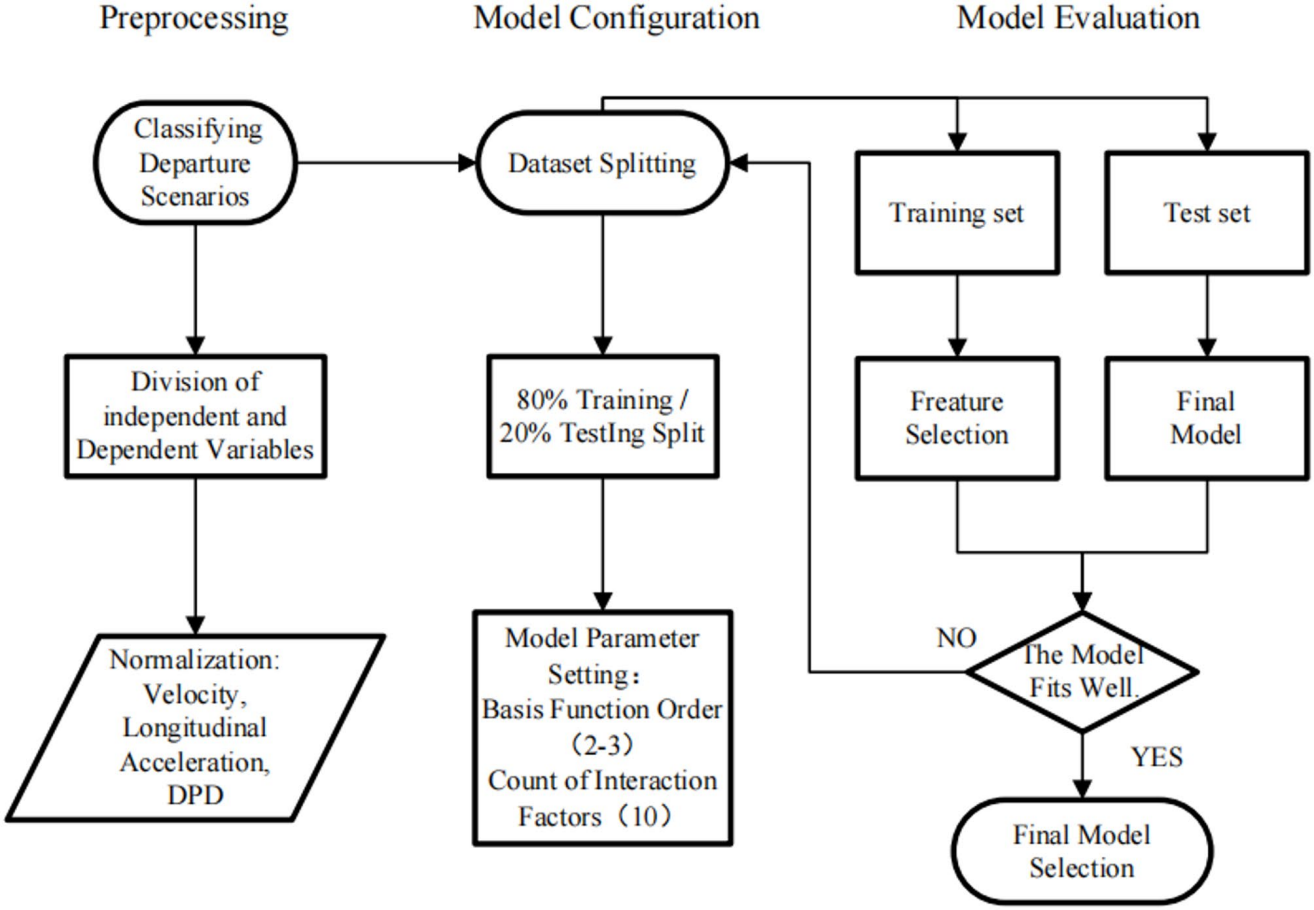
Flowchart of the MARS model construction.

After constricting the base functions, variables with low correlations with the dependent variable were removed based on their correlation coefficients. Next, the best-fitting model generated corresponding constant terms, base functions. Finally, the performance of the model was evaluated using the test data set, and several evaluation parameters, including Generalized Cross-Validation (GCV), Residual Sum of Squares (RSS), R-squared (RSq), Global R-squared (GRSq), Mean Squared Error (MSE), were obtained. The entire model building process is shown in Fig. 10.

### Model construction

The MARS model was used to study the *maximum lateral departure* (MLD) of the four different combined curves separately. After separation by centrifugal force direction, there were a total of 8 datasets, each corresponding to a specific model-with these metrics derived from the test dataset. The predictive performance, training mean squared error, and testing mean squared error of these 8 MARS models in *maximum lateral departure* (MLD) is separately shown.

The eight maximum lateral departure models’ performance metrics of the four types of combined curves are shown in the Table 6.

**Table 6:** The maximum lateral departure models’ performance metrics of the four types of combined curves.

Curve type	Model type	GCV	RSS	GRSq	RSq	Train MSE	Test MSE
Downslope-curve	IDCF	0.68	42.96	0.33	0.57	0.43	0.79
	ADCF	0.69	49.57	0.32	0.55	0.45	0.57
Upslope-curve	IDCF	0.62	45.47	0.38	0.57	0.42	0.55
	ADCF	0.76	66.21	0.25	0.38	0.61	0.58
Sag-curve	IDCF	0.89	83.34	0.11	0.17	0.83	0.68
	ADCF	0.60	25.65	0.41	0.68	0.31	0.40
Crest-curve	IDCF	0.83	50.36	0.18	0.29	0.70	0.40
	ADCF	0.63	26.81	0.38	0.64	0.36	0.85

Taking the *maximum lateral departure* (MLD) model results for the IDCF departure event on downslope-curve scenario as an example. The Generalised Cross-Validation (GCV) is 0.68, which is relatively low among all model-scenario combinations, indicating excellent predictive performance of the model. This signifies that the model has strong adaptability to unseen data while effectively balancing the complexity of the model structure, thereby significantly mitigating the risk of overfitting. The Residual Sum of Squares (RSS) is 42.96, one of the smallest RSS values in the table, suggesting that the model’s predictions of *maximum lateral departure* (MLD) are highly consistent with the observed actual data, reflecting a high degree of fitting accuracy. The Global R-Squared (GRSq) is 42.96, a moderately high value, indicating that the model possesses strong overall explanatory power for the dependent variable (MLD).

Notably, the R-squared (RSq) value reaches 0.57. This higher RSq demonstrates the model’s exceptional capacity to explain a large proportion of the variability in the dependent variable, thereby underscoring its outstanding predictive strength. Additionally, A training MSE of 0.43 indicates a good fit to the underlying data patterns, while a test MSE of 0.79 reflects competent generalization to unseen data. The relatively small gap between these two values signifies that the model has not overfit and possesses good stability. This conclusion is further strengthened by the low Generalized Cross-Validation (GCV) score, collectively affirming the model’s reliability and practical utility.

The goodness of fit of the MARS modelling for the *maximum lateral departure* (MLD) for all combined curves is satisfactory, and therefore the study decided to focus primarily on the MARS models built with *maximum lateral departure* (MLD) as the dependent variable. The *departure duration distance* (DPD) is used as a dependent variable in the modelling process. The MARS models for the four types of combined curves are analyzed separately.

## Modeling results and interpretation

### In the direction of centrifugal force (IDCF)

To investigate the extent and mechanisms of *maximum lateral departure* (MLD) under different horizontal–vertical curve combinations, MARS models were constructed for IDCF scenarios across four curve types: downslope, upslope, sag, and crest. The *maximum lateral departure* (MLD) model under IDCF conditions is summarized in the table below. The magnitude of the coefficients reflects the contribution of these terms to the model.

Table 7 summarizes the key basis functions and coefficients of the MLD models under IDCF conditions. Each model incorporates a combination of driver characteristics, speed variables, and derived interaction terms. These results serve as the foundation for identifying dominant predictors and nonlinear behavior patterns, which are further analyzed in the following subsections.

**Table 7:** Summary of IDCF MLD models.

Curve type	Term	Basis function	Coefficient	Variable	Description
Downslope-curve	Intercept	Constant	0.581		
	T1	h (Age − 3)	− 0.652	Age	Age
	T2	h (CV11 − 2)	1.978	CV11	Mountainous freeway driving frequency
	T3	h (CNV1 − 0.942172)	7.612	CNV1	Average speed
	T4	h (DPD − -0.432384)	− 1.669	DPD	Departure Duration Distance
	T5	CV9 × T3	− 1.061	CV9	Average daily driving distance on workdays
	T6	CV5 × T4	1.697	CV5	Driving experience on mountainous freeways
	T7	DPD × T3	3.456	DPD	Departure Duration Distance
	T8	h (6 − CV8) × h (DPD − -0.528463)	− 0.462	CV8	Average daily driving time on workday
Upslope-curve	Intercept	Constant	0.292		
	T1	h (DPD − -0.394924)	− 2.787	DPD	Departure Duration Distance
	T2	h (DPD − -0.0998095)	2.625	DPD	Departure Duration Distance
	T3	Gender1 × h (-0.611965 − CNV1)	2.403	Gender	Gender
	T4	CV3 × h (-0.611965 − CNV1)	1.001	CV3	Years of driving experience
	T5	CV7 × h (-0.611965 − CNV1)	0.266	CV7	Driving license type
	T6	CV11 × h (-0.611965 − CNV1)	− 1.899	CV11	Mountainous freeway driving frequency
Sag-curve	Intercept	Constant	− 0.509		
	T1	h (0.760099 − DPD)	0.543	DPD	Departure Duration Distance
Crest-curve	Intercept	Constant	− 0.437		
	T1	h (-0.0558893 − DPD)	2.516	DPD	Departure Duration Distance
	T2	CV10 × T1	− 0.347	CV10	Average kilometers driven in the past year

#### Key predictors of IDCF models

The IDCF models reveal that the main predictors of *maximum lateral departure* (MLD) are strongly dependent on the specific horizontal–vertical curve combination. *Departure duration distance* (DPD) remains a crucial, highly nonlinear predictor across all four curve types, while average speed (CNV1) emerges as a particularly potent factor in the downslope model.

*Departure duration distance* (DPD): A significant predictor across all four curve models, demonstrating a consistent yet complex nonlinear association with *maximum lateral departure* (MLD). In the upslope-curve model, its effect is piecewise, imply that *maximum lateral departure* (MLD) initially decreases as *Departure duration distance* (DPD) above 45 m (transformed value: -0.395), but increases sharply once *Departure duration distance* (DPD) exceeds a critical threshold of approximately 65 m (transformed value: -0.10). This pattern underscores the elevated risk associated with prolonged departures. Conversely, in the downslope-curve model, the negative coefficient (-1.669) of h(DPD − -0.432384), indicates higher *maximum lateral departure* (MLD) when *Departure duration distance* (DPD) is shorter than the threshold of about 40 m (transformed value: -0.43).

Average speed (CNV1): Average speed is a major factor of downslope-curve model in the IDCF scenario, with a large positive coefficient of 7.612). This term indicates that *maximum lateral departure* (MLD) increases dramatically when the average speed exceeds 108.63 km/h (0.942), suggesting that high vehicle speed in downslope curves is the most significant standalone factor contributing to extreme lateral departure.

Mountainous freeway driving frequency (CV11): This variable exhibits contrasting effects: This variable exhibits contrasting effects. In the downslope-curve model, high mountainous freeway driving frequency has a large positive coefficient (1.978), indicating that frequently driving on mountainous freeways is associated with higher *maximum lateral departure* (MLD) on downslope sections. This might point to drivers adopting riskier habits or becoming overconfident. Conversely, in the upslope-curve model, high frequency interacts with low speed with a negative coefficient (-1.899), suggesting that under slow-speed, upslope conditions, frequent exposure to mountain roads is protective against high *maximum lateral departure* (MLD).

**Fig. 11:**
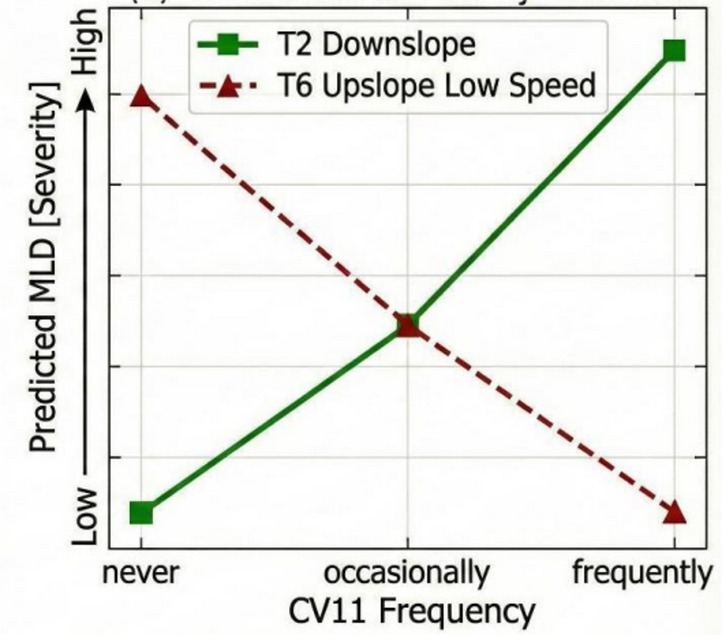
Influence of mountainous freeway driving frequency (CV11) on maximum lateral departure (MLD) under IDCF conditions.

As shown in Fig. 11, mountainous freeway driving frequency (CV11) had a notable effect on *Maximum lateral departure* (MLD) under both downslope and upslope scenarios. Downslope-curve: As the CV11 frequency increases, the *maximum lateral departure* (MLD) shows an upward trend, shifting from low risk to high risk. Upslope-curve interaction with low speed (lower than 86.77 km/h): As the CV11 frequency increases, the *maximum lateral departure* (MLD) shows a downward trend, shifting from high risk to low risk, clearly demonstrating the protective effect of CV11 under low-speed upslope-curve conditions.

Age and Gender: Age has a negative main effect (-0.652) in the downslope-curve model, implying that higher age (above 49 years old) is associated with lower *maximum lateral departure* (MLD), suggesting better control or more conservative driving on downslopes. Gender (male) only appears as an interaction term in the upslope-curve model, indicating its influence is strictly contextual.

Compared to these, other factors like average daily driving distance on workdays (CV9) and driving experience on mountainous freeways (CV5) play more specific, localized roles within individual curve models.

#### Representative interactions in IDCF models

In addition to the main effects, several interaction terms in the IDCF models provided further insights into how combinations of driver characteristics affect lane departure behaviors. The interaction terms reveal critical synergistic and conditional effects, demonstrating how driver characteristics amplify or mitigate risks under specific road conditions.

Interaction of driver exposure and speed in downslope-curve (T5 and T7):

The interaction between average daily driving distance on workdays (CV9) and the high-speed term (CNV1) is negative (-1.061). This means that the extreme risk posed by high speed is mitigated for drivers who drive long distances daily. High driving exposure might equate to better vehicle handling or more practiced corrective responses, even under high-speed conditions. Conversely, the interaction between *departure duration distance* (DPD) and the high-speed term is strongly positive (3.456). This underscores a compounding risk: when the high-speed condition (speed > 108.63 km/h) is met, any extension of the *departure duration distance* (DPD) rapidly amplifies the *maximum lateral departure* (MLD) to an even greater degree.

Interaction of experience and *departure duration distance* (DPD) in downslope-Curve (T6):

Driving experience on mountainous freeways (CV5) interacts with the *departure duration distance* (DPD) (T4) with a positive coefficient (1.697). When the *departure duration distance* (DPD) exceeds 40.00 m, indicating a slight reduction in risk (as the T4 term lowers the *maximum lateral departure* (MLD)). However, drivers with mountain driving experience (CV5) partially offset the protective effect of T4, thereby increasing the *maximum lateral departure* (MLD). This might suggest that experienced mountain drivers make more assertive, less contained maneuvers or have higher speed expectations that lead to greater initial departure.

Interaction of experience and low speed in upslope-curve (T4 and T5):

In the upslope-curve, several terms (Gender, CV3, CV7, CV11) interact with the speed lower than 86.77 km/h (-0.611965). The positive coefficients for years of driving experience (CV3) (1.001) and driving license type (CV7) (0.266) indicate that under potentially slow speed conditions, higher general experience and lower grade license types are associated with higher *maximum lateral departure* (MLD). This may suggest these experienced drivers become impatient or attempt more aggressive passing/corrective maneuvers during slow-speed lane departure events, leading to greater lateral departure.

Drivers with lower-level licenses (such as those for small vehicles, lacking the specialized training required for large vehicles) are associated with higher *maximum lateral departure* (MLD).

Contrasting predictors in crest-curve (T2): In the crest-curve model, where T1 indicates a positive *maximum lateral departure* (MLD) effect for short *departure duration distance* (DPD) (shorter than 40.0 m), the interaction with average kilometers driven in the past year (CV10) (T2) has a negative coefficient (-0.347). This suggests that high recent driving mileage acts as a protective factor, mitigating the initial lateral departure associated with brief lane departures on crest curves.

These interactions are crucial for identifying which combinations of driver characteristics and road conditions create the most significant risk profiles for lateral departure.

The identified nonlinear threshold behaviors of *departure duration distance* (DPD) and CNV1 (speed) are further synthesized and visualized in Sect. 7.

### Against the direction of centrifugal force (ADCF)

Compared to IDCF, ADCF behaviors reflect a different driver response mode that involves more subtle counter-steering and higher sensitivity to crash-related experience. The *maximum lateral departure* (MLD) model under ADCF conditions is summarized in the table below (Table 8).

**Table 8:** Summary of ADCF MLD models.

Curve type	Term	Basis function	Coefficient	Variable	Description
Downslope-curve	Intercept	Constant	− 0.074		
	T1	h (CV7 − 6)	− 0.875	CV7	Driving license type
	T2	h (2 − CV9)	− 1.470	CV9	Average daily driving distance on workdays
	T3	− T2	− 0.180	CV9	Average daily driving distance on workdays
	T4	h (CV9 − 8)	4.364	CV9	Average daily driving distance on workdays
	T5	h (1.05138 − DPD)	0.364	DPD	Departure Duration Distance
	T6	CV6 × h (2 − CV11)	0.463	CV6	Road expert type
	T7	T4 × CV4	− 0.964	CV4	Freeway driving frequency
	T8	T5 × h (2 − CV11)	− 1.667	CV11	Mountainous freeway driving frequency
Upslope-curve	Intercept	Constant	− 1.191		
	T1	CV5	0.913	CV5	Driving experience on mountainous freeways
	T2	h (2 − CV10)	− 1.633	CV10	Average kilometers driven in the past year
	T3	CV8 × T2	0.629	CV8	Average daily driving time on workday
	T4	h (CV3 − 2) × h (CV7 − 5)	1.346	CV3	Years of driving experience
Sag-curve	Intercept	Constant	− 0.541		
	T1	h (CV7 − 6)	2.257	CV7	Driving license type
	T2	h (2 − CV6)	1.996	CV6	Road expert type
	T3	h (7 − CV9)	− 1.480	CV9	Average daily driving distance on workdays
	T4	h (2 − CV4)	1.696	CV4	Freeway driving frequency
	T5	h(2 − CV11)	1.143	CV11	Mountainous freeway driving frequency
	T6	h (CNV1 − 0.611089)	0.710	CNV1	Average speed
	T7	CV3 × T3	0.255	CV3	Years of driving experience
	T8	CV6 × T3	0.333	CV6	Road expert type
	T9	CV8 × T3	0.121	CV8	Average daily driving time on workday
Crest-curve	Intercept	Constant	− 0.722		
	T1	h (2 − CV6)	− 1.658	CV6	Road expert type
	T2	−T1	− 3.203	CV6	Road expert type
	T3	h (CV9 − 7)	3.438	CV9	Average daily driving distance on workdays
	T4	h (2 − CV11)	1.633	CV11	Mountainous freeway driving frequency
	T5	h (CV6 − 2) × CV14	1.631	CV14	Driving frequency
	T7	T3 × CV6	− 1.426	CV6	Road expert type
	T8	CV15 × h (-0.310353 − DPD)	0.285	CV15	Types of vehicles usually driven

Table 8 presents the full model structures for ADCF across the four curve types, highlighting both main effects and interaction terms. Based on these models, we identified critical influencing factors such as crash history, driving exposure, and experience levels. The following analysis discusses these predictors and interactions in detail.

#### Key predictors of ADCF models

The key predictors of *maximum lateral departure* (MLD) in the ADCF scenario are highly concentrated in driver exposure, expertise, and license type, indicating that the ability to successfully counter-steer is heavily reliant on specific driver demographics.

Average daily driving distance on workdays (CV9) emerges as the most influential yet complex factor, predominantly impacting the downslope-curve and crest-curve models. Its relationship with *maximum lateral departure* (MLD) is characterized by a piecewise function within the downslope model. For low-mileage drivers (daily distance < 6 km), carries a strongly negative coefficient (-1.470). This indicates a significantly lower *maximum lateral departure* (MLD), which may be attributed to heightened caution and minimal lateral departure during driving.

**Fig. 12:**
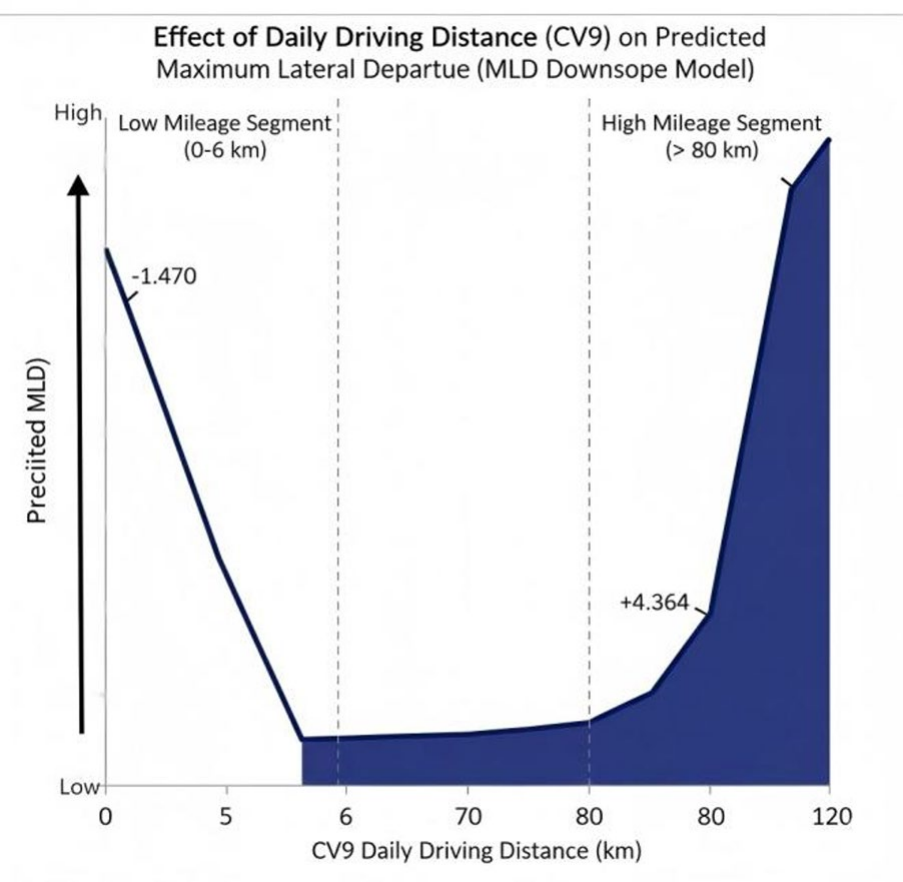
Average daily driving distance on workdays (CV9) effect MLD under ADCF conditions.

Conversely, for high-mileage drivers (daily distance > 80 km), with its substantial positive coefficient (4.364), becomes active. This association with elevated *maximum lateral departure* (MLD) suggests that extensive driving exposure might lead to more pronounced or abrupt corrective maneuvers (ADCF), consequently resulting in larger lateral departures. Overall, the analysis reveals a profoundly nonlinear relationship, where *maximum lateral departure* (MLD) peaks at the highest levels of driving exposure. As shown in Fig. 12.

Driving license type (CV7) is a strong predictor in the downslope-curve and sag-curve models. On the downslope-curve has a negative coefficient (-0.875). The negative coefficient suggests that lower license categories (small automatic cars) are generally associated with lower *maximum lateral departure* (MLD), possibly due to these curves are simpler and making it easier for drivers to control the vehicle. On the sag-curve has a large positive coefficient (2.257). This shows that the drivers with small automatic cars license is associated with significantly higher *maximum lateral departure* (MLD) on sag curves. This may be due to drivers with licenses for small automatic vehicles lacking experience in complex road conditions, as such vehicles exhibit less stability than larger vehicles when subjected to vertical force changes while navigating concave curves.

The road expert type (CV6) acts as a critical moderator across several models, highlighting the value of safety expertise. On the crest-curve model, there has a strongly negative coefficient (-1.658). This means that when the driver is not a road expert (category < 2)—i.e., not a planner or safety technician—*maximum lateral departure* (MLD) is significantly lower. This is counter-intuitive and suggests that in this specific crest-curve ADCF scenario (corrective steering on a crest), non-experts might be more tentative, leading to smaller *maximum lateral departure* (MLD), while experts might be overconfident or correct more aggressively. On the sag-curve, there has a positive coefficient (1.996), confirming that being a non-expert is associated with higher *maximum lateral departure* (MLD) on sag curves. This aligns with the expectation that expertise is protective on complex curves.

*Departure duration distance* (DPD): with a positive coefficient (0.364) appears in the downslope-curve model. The corresponding threshold for ADCF is 110.00 m (1.051). Interpretation: when the *departure duration distance* (DPD) is less than 110.00 m, the *maximum lateral departure* (MLD) increases as the *departure duration distance* (DPD) increases. This confirms that in ADCF events on downslope-curves, short *departure duration distance* (DPD) departure are associated with the largest magnitude of lateral displacement, likely due to rapid, sudden counter-steering.

#### Representative interactions in ADCF models

The interaction terms reveal critical synergistic effects, often amplifying the risk associated with high-exposure or non-expert status.

The ADCF models revealed more complex interaction effects, primarily involving *departure duration distance* (DPD), road expert (CV6), and driving experience (CV4, CV9, CV11, CV15). These interactions offer deeper insights into how specific driver groups respond under counter-directional departure scenarios in combined alignments.

**Fig. 13:**
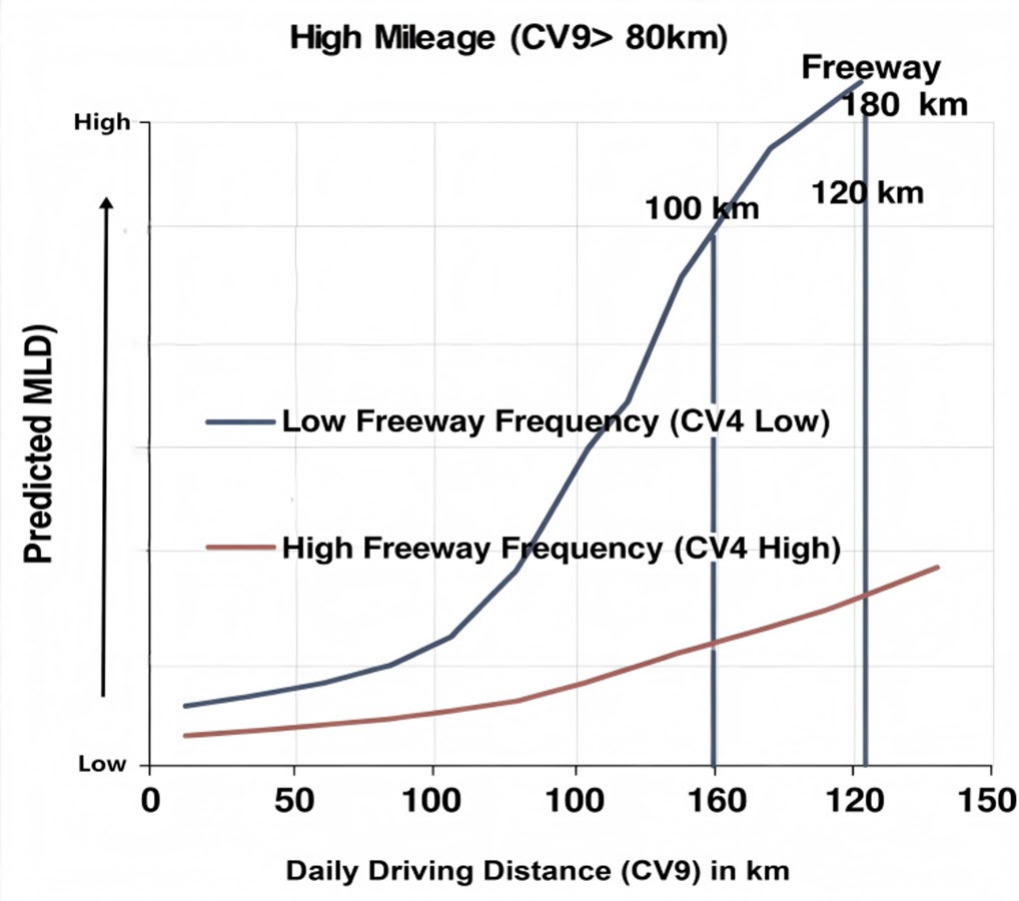
High mileage and freeway frequency interaction effects on MLD under ADCF models.

Interaction of high mileage and freeway frequency in downslope-curve: with a negative coefficient (-0.964). Recall that high mileage is the high-risk term for drivers over 80 km daily (CV9 > 8). Interpretation: The negative interaction indicates that the *maximum lateral departure* (MLD) risk posed by very high daily mileage is significantly mitigated for drivers with higher freeway driving frequency (CV4). This suggests that while driving long distances is inherently risky, having high overall freeway exposure provides a protective, moderating effect on the magnitude of the counter-steering departure. As shown in Fig. 13.

Interaction of road expert type and low mountain frequency in downslope-curve: with a positive coefficient (0.463). h (2 − CV11) is active when mountainous freeway driving frequency (CV11) is “never”. Interpretation: being a road expert (CV6 high) combined with never driving on mountain freeways leads to higher *maximum lateral departure* (MLD). This highlights a lack of specific terrain experience for experts, which reduces their ability to execute successful counter-steering on downslopes, potentially due to over-reliance on theoretical knowledge rather than practical terrain experience.

In contrast, a significant negative interaction was observed between driver expertise and high mileage in crest curves, with a negative coefficient (-1.426). the main effect of high daily mileage (driving over 60 km daily on workdays, CV9 > 7) is associated with increased risk. Interpretation: the negative interaction shows that being a road expert (CV6 = 2, 3) significantly reduces the high *maximum lateral departure* (MLD) risk associated with high daily mileage on crest curves. This confirms that at the high levels of driver exposure, safety expertise acts as a crucial protective factor, improving counter-steering control near the crest.

**Fig. 14:**
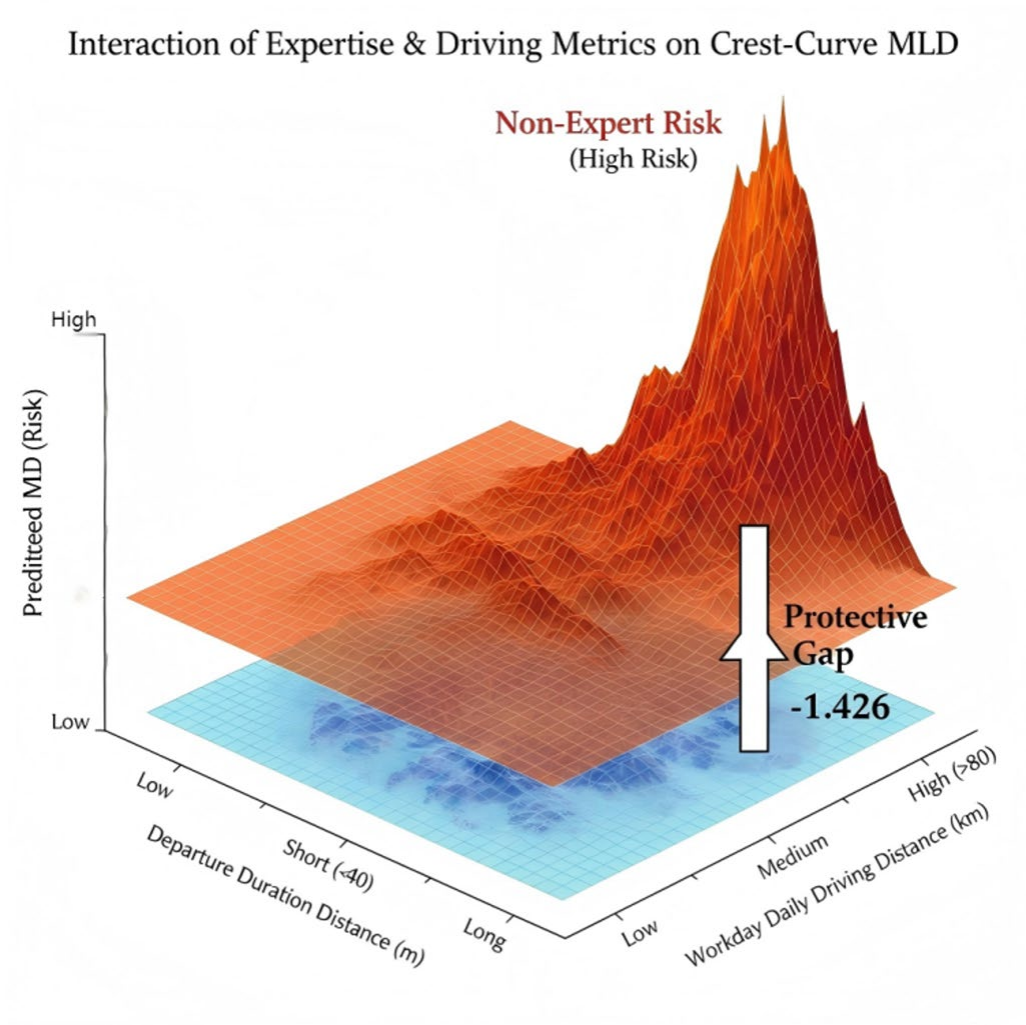
Interaction of road expert & driving metrics on crest-curve.

These relationships are visually summarized in the Fig. 14, through a three-dimensional surface plot, the protective effect of the “Road Expert” status against *maximum lateral departure* (MLD) risks on crest-curve sections under varying conditions of daily driving distance per workday (CV9) and *departure duration distance* (DPD). Among them, the upper plane represents the risk profile for non-expert drivers. As daily driving distance increases, this plane rises sharply, forming a pronounced peak of non-expert risk. The lower plane represents the risk profile for road experts. This plane remains a gentle safety plateau, showing no significant increase even in high-mileage zones.

Types of vehicles usually driven and short *departure duration distance* (DPD) in crest-curve: with a positive coefficient (0.285). The main interaction effect of types of vehicles usually driven and short *departure duration distance* (DPD) (less than 40.00 m) is associated with increased risk. Interpretation: the result shows that drivers who usually drive manual transmission vehicles (CV15 = 2) exhibit higher *maximum lateral departure* (MLD) than automatic transmission drivers (CV15 = 1) during rapid corrective events on a crest curve. This effect is attributed to habit transfer. Drivers accustomed to manual transmission are likely to possess a more aggressive or direct control style, leading them to input larger, faster steering corrections in the car. This tendency for over-correction in high-pressure, low-visibility scenarios (crest curves) results in a significantly larger *maximum lateral departure* (MLD).

Overall, the driving experience, road expert type, and *departure duration distance* (DPD) had significantly influence on departure severity. Also, driver characteristics and lane *departure duration distance* (DPD) had an interaction effect on departure severity.

These interaction patterns confirm that driver background, experience type, and real-time vehicle dynamics interact in complex ways to determine lane departure severity. The nonlinear threshold behaviors of key variables like average speed (CNV1) are further synthesized in Sect. 7.

## Thresholds for lane departure

These interactions underscore that ADCF events are not merely caused by individual risk factors, but rather by compound effects of experience, driving group, and driving exposure in complex geometric environments.

For the IDCF and ADCF modelling results of the four types of combined curves, some thresholds for *departure duration distance* (DPD) and average speed are obtained, which can be used as a basis for ADAS systems, road design, or driver-targeted training. Since both *departure duration distance* (DPD) and average speed are normalized during modelling, here we backtrack to calculate the original values.

### Departure duration distance

**Fig. 15:**
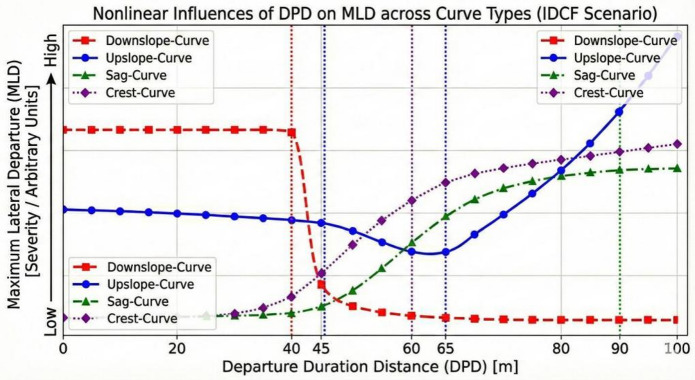
Departure duration distance across curve types (IDCF scenario).

*Departure duration distance* (DPD) thresholds mark critical transition points in driver behavior: Below threshold values indicate escalating risk requiring early warnings, while exceeding thresholds often triggers corrective actions. Significant differences exist between IDCF and ADCF responses. *Departure duration distance* (DPD) thresholds for IDCF as shown in Fig. 15.

The *departure duration distance* (DPD) serves as a fundamental indicator of sustained departure risk, with its influence mechanism being highly asymmetric across different intervals and curve types.

For the IDCF scenario, the following observations were obtained:

Downslope-curve: The 40.00 m (-0.432) is a critical threshold. *Maximum lateral departure* (MLD) is notably high for brief *departure duration distance* (DPD) (shorter than 40.00 m). Beyond this distance, the T4 term (h (DPD − -0.432384)) with a negative coefficient (-1.669) activates, suggesting that longer, more sustained departure are associated with lower overall *maximum lateral departure* (MLD). This highlights the inherent risk in rapid, initial steering instability. Furthermore, driving experience on mountainous freeways (CV5) interacts with this term (T6: 1.697), showing that specialized experience amplifies *maximum lateral departure* (MLD) when *departure duration distance* (DPD) is beyond 35.00 m, possibly due to more aggressive or delayed corrections.

Upslope-curve: *Departure duration distance* (DPD) exhibits a piecewise risk structure, with thresholds at 45.00 m (-0.395) and 65.00 m (-0.100). When a vehicle departure for a short *departure duration distance* (DPD) (greater than 45 m and less than 65 m), the driver may be experiencing an initial distraction or momentary lapse. In such cases, the driver promptly detects the departure and initiates effective lane-keeping corrective actions, leading to a sharp decline in *maximum lateral departure* (MLD). When the *departure duration distance* (DPD) exceeds 65.00 m (T2: 2.625). This indicates that on upslope-curves, sustained time outside the lane boundary is a critical amplifier of lateral departure severity.

Sag-curve: The 90.00 m (0.760) is a threshold, where the degree of IDCF departure increase with increasing distance for *departure duration distance* (DPD) below 90.00 m (T1: 0.543). This suggests that short *departure duration distance* (DPD) are the primary source of high *maximum lateral departure* (MLD) risk on sag curves.

Crest-curve: The 60.00 m (-0.056) is the key threshold. T1 (2.516) indicates that when the *departure duration distance* (DPD) is less than 60.00 m, the *maximum lateral departure* (MLD) increases as the *departure duration distance* (DPD) increases. This initial high risk is subject to driver exposure: the *maximum lateral departure* (MLD) risk is increased as the *departure duration distance* (DPD) decrease for drivers with high average kilometers driven in the past year (CV10) (T2: -0.347), but this benefit diminishes as the departure extends beyond 60.00 m.

**Fig. 16:**
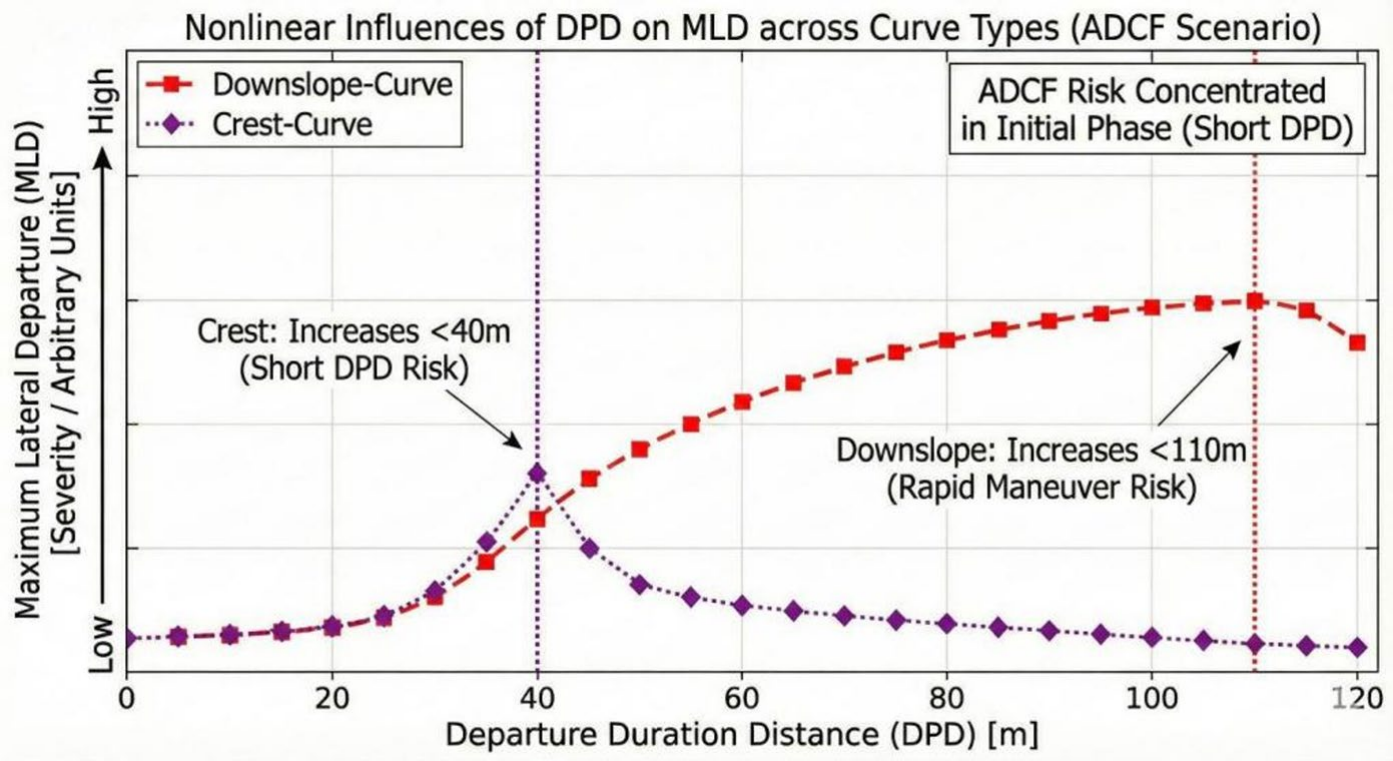
Departure duration distance across curve types (ADCF scenario).

*Departure duration distance* (DPD) thresholds for ADCF as shown in Fig. 16.

The effect of *departure duration distance* (DPD) on ADCF departure differs from that on IDCF departure.

Downslope-curve: The 110.00 m (1.051) is a threshold (T5: h (1.05138 − DPD), 0.364). The degree of lane departure increases with increasing distance when *departure duration distance* (DPD) below 110.00 m. This confirms that rapid counter-steering or quick lane change maneuvers (short DPD) lead to greater lateral displacement against the centrifugal force.

Crest-curve: The 40.00 m (-0.310) is the critical threshold. When the *departure duration distance* (DPD) is less than 40.00 m, the *maximum lateral departure* (MLD) increases as the *departure duration distance* (DPD) increases. High-mileage drivers (CV10) interact with this term (T6: -1.118), leading to a decreased degree of ADCF departure, demonstrating greater precision in short *departure duration distance* (DPD) spatial judgment. Conversely, usually driving an automatic transmission vehicle (CV15) interacts with this term (T8: 0.285), which increases *maximum lateral departure* (MLD). This reflects the greater magnitude of lateral departure associated with the habitual control style of automatic drivers during rapid corrections.

Collectively, this demonstrates that for ADCF events, the risk is predominantly concentrated in the initial phase of the departure (short DPD), where driver experience, and established vehicle control habits determine the magnitude of the corrective overshoot.

The *departure duration distance* (DPD) limit values were calculated for the four combination curves. Therefore, 35.00 m (-0.528) can be used as a warning threshold to indicate the presence of a slight departure in the on-board system and to suggest steering wheel adjustment. 110.00 m (1.051) can be used as an “emergency threshold” to trigger active safety systems (e.g., automatic braking or steering assistance).

### Average speed

**Fig. 17:**
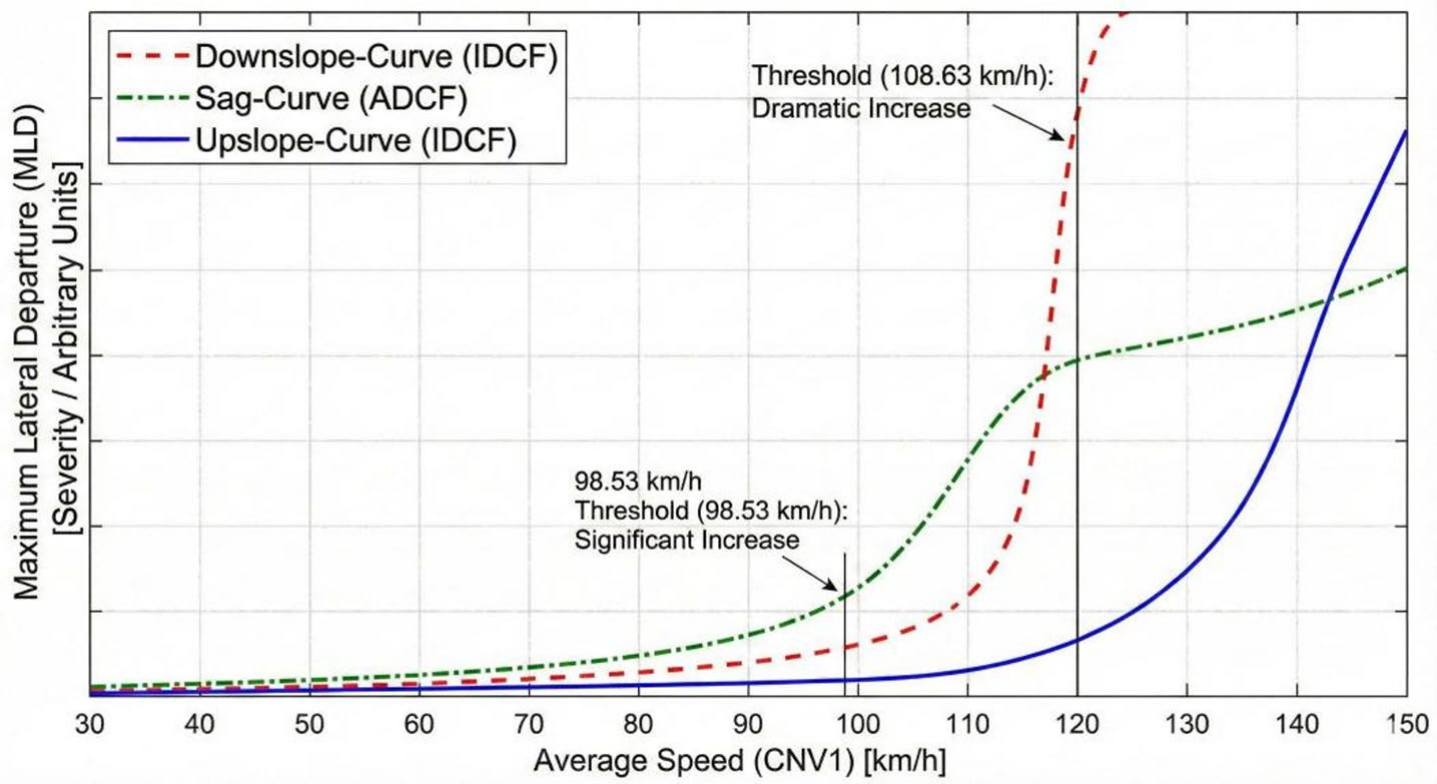
Speed thresholds across curve types.

Average speed affects sag-curves ADCF departure significantly, also significantly affects the upslope-curves and downslope-curves of the IDCF departure. As shown in Fig. 17.

Average speed (CNV1) remains a dominant predictor, with profound nonlinear effects defining safe operational speed. Downslope-curve (IDCF): average speed is an exceptionally dominant factor (T3: 7.612) with a threshold at 108.63 km/h (0.942). The *maximum lateral departure* (MLD) increases dramatically when the average speed exceeds 108.63 km/h. This indicates that high speed control is the critical determinant of vehicle trajectory stability on downslope-curves. This high-speed risk is amplified by the *departure duration distance* (DPD) of the departure (T7: 3.456) but slightly mitigated by high daily driving exposure (T5: -1.061).

Sag-curve (ADCF): average speed (CNV1) influences ADCF departure through a single basis function with a threshold at 98.53 km/h (0.611) (T6: 0.710). The *maximum lateral departure* (MLD) risk increases as speed exceeds this threshold. This suggests that while sag curves are primarily a vertical challenge, excessive speed significantly compromises the driver’s ability to counter-steer effectively against lateral departure.

Overall, the *departure duration distance* (DPD) of the IDCF and ADCF model is significant; the average speed (CNV1) variable in the sag-curve of the ADCF model and the downslope-curve and up-curves of the IDCF model is significant. The thresholds are summarized in the Table 9.

**Table 9:** The departure duration distance and average speed thresholds summarize.

Type	IDCF				ADCF			
Variable	Down slope-curve	Upslope-curve	Sag-curve	Crest-curve	Down slope-curve	Upslope-curve	Sag-curve	Crest-curve
Departure duration distance	35.00 m (− 0.528) 40.00 m (− 0.433)	45.00 m (− 0.395) 65.00 m (− 0.100)	90.00 m (0.760)	60.00 m (− 0.055)	110.00 m (1.051)	–	–	40.00 m (− 0.310)
Average speed	108.63 km/h (0.942)	86.77 km/h (− 0.612)	–	–	–	–	100.20 km/h (0.661)	

## Discussions

In this study, the multiple adaptive regression spline (MARS) model was utilized to provide an in-depth analysis of the IDCF departure and ADCF departure for four types of combination curves. Compared with the traditional linear regression model, the application of the MARS model effectively captures the nonlinear relationship between driver characteristics, lane departure during distance and vehicle speed on the degree of lane departure. The results of the study are consistent with some existing studies. The results and thresholds of the lane departure behaviour model can reflect the drivers’ risk perception and corrective ability.

Road Experts (CV6): road experts (traffic planners, safety technicians) exhibit contradictory lane departure behaviors. In the sag-curve ADCF model, being a non-expert (CV6 < 2) is a significant risk factor, suggesting expert knowledge is protective where vertical geometry is complex. Conversely, in the crest-curve ADCF model, the expert status interacts with high daily mileage to provide a significant protective moderation, indicating superior control capability when exposure risk is high. This duality emphasizes that expert knowledge provides superior risk mitigation against high exposure (crest/high mileage) but is fundamentally essential for maintaining stable lateral control in complex vertical geometry (sag-curves).

License type (CV7) is a powerful but contradictory predictor, reflecting both the quality of training and the associated vehicle class. In the downslope-curve ADCF model, higher license grades (lower CV7 code) act as a strong protective factor, suggesting superior training associated with complex vehicle handling yields benefits. However, in the sag-curve ADCF model, the lowest license grades (CV7 = 6, small automatic cars) are associated with significantly higher *maximum lateral departure* (MLD). This finding indicates that mere legal driving privilege is insufficient; professional training and experience navigating vehicle dynamics under complex vertical gradients are crucial to mitigating lateral instability.

The impact of accumulated driving exposure is highly complex, showing severe nonlinear effects.

Average daily driving distance (CV9): The effect of daily mileage is extremely nonlinear in the downslope-curve ADCF model. Low mileage drivers (less than 6 km daily) exhibit low *maximum lateral departure* (MLD), likely due to high caution. However, very high mileage drivers (over 80 km daily) transition into an extremely potent risk factor. This suggests that high exposure, while fostering proficiency, also readily cultivates “risk perception dullness” or overconfidence, leading to aggressive steering operation (ADCF) that result in lateral departure. However, this risk is, partially mitigated when combined with high general freeway driving frequency (CV4), suggesting general freeway exposure provides a corrective buffer.

Types of vehicles usually driven (CV15): The type of vehicle usually driven (CV15) significantly affects lane departure behavior during rapid corrections in the crest-curve ADCF model. Manual transmission drivers show higher *maximum lateral departure* (MLD) compared to automatic drivers. This result is explained by habit transfer: manual drivers may develop a more direct, forceful, or aggressive control style that translates into larger, over-corrected steering inputs in high-pressure scenarios, thus increasing *maximum lateral departure* (MLD). This increased lateral departure following an initial departure event can be explained by the risk compensation theory^72^. High-risk drivers may overcorrect the steering wheel at short departure, and may compensate for the initial slight departure by risky maneuvers (e.g., cutting corners), leading to an increase in the *maximum lateral departure* (MLD).

Specialized experience (CV5): driving experience on mountainous freeways (CV5) consistently acts as a risk factor in the upslope-curve ADCF model. This indicates that while specialized experience hones a driver’s anticipation skills, it may also lead to habitual risk adaptation, where drivers use their competence to push vehicle control limits in complex environments, resulting in higher lateral departure during corrections. The result highlights that simple accumulation of time and experience does not guarantee safety; the quality and context of that experience are paramount.

Years of driving experience (CV3): The role of driving experience is highly contextual. In certain scenarios, such as low-speed conditions on the upslope-curve ADCF model, higher years of experience actually acts as a risk factor. This suggests that accumulated years, especially if not translated into high-quality specialized exposure, may backfire by reinforcing poor driving habits or cultivating impatience, leading to more aggressive corrective maneuvers under slow, complex conditions. This finding further validates the conclusion that mere years on the road offer limited protection unless refined by high-quality specialized experience^73^.

Meanwhile, the interaction between road geometric design and driving behaviour is reflected. 1) It is found that the average frequency of lane departure behaviour is higher in sag-curves and crest-curves than downslope-curves and upslope-curves. 2) The *departure duration distance* threshold of downslope-curve, 110.00 m is significantly longer than that of other combined curves; meanwhile, sa*g*-curve, where the degree of lane departure increases as the *departure duration distance* (DPD) increases when the *departure duration distance* (DPD) is less than 90.00 meters. The vehicle inertia is enhanced, and the difficulty of correcting the departure continues to increase. The higher driving complexity (speed-selective behaviour) of sag-curve may be due to the drivers’ visual mutation or perceived centrifugal force mutation^47^. These results suggest support for Lamm’ s driver perception hypothesis^74^, also noted by Bella^56,75^, that an overlapping crest-curve may cause the horizontal curve to look sharper than it is, while a sag-curve may cause the horizontal curve to look sharper than it is. while a sag-curve may cause the horizontal curve to look flatter.

The effect of vehicle speed on lane departure behaviour is demonstrated in this study. In particular, the degree of ADCF lane departure increases significantly for sag-curve when speed exceeds the threshold. Also, the degree of IDCF lane departure increases significantly for upslope-curve and downslope-curve with speeds above a threshold.

The low threshold of *departure duration distance* (35.00 m) may reflect the drivers’ unconscious corrective ability (i.e., habitual operation); the high threshold (110.00 m) may trigger the drivers’ passive stress response, which makes drivers take more aggressive actions.

Compared with other studies, this research pass delves into the nuanced relationship between driver characteristics, roadway type, and the two types of lane departure behaviours. Driver characteristics not only affect the frequency of lane departure, but also the directionality and degree of departure, which provides new perspectives for future road design and driving safety research, revealing how drivers adopt different coping strategies in the two types of lane departure under different driving styles.

## Conclusion

Based on driving simulation experiments, this study investigates the effects of driver characteristics and four types of combined curves on lane departure behaviour. Based on the direction of centrifugal force, the study classified the lane departure behaviour into two categories: *In the Direction of Centrifugal Force* (IDCF) *and Against the Direction of Centrifugal Force* (ADCF). And eight models were built for IDCF and ADCF respectively. The model results revealed the significant effects of driver characteristics on lane departure behaviour (IDCF and ADCF) on combined curves. Thresholds for lane departure duration distance and vehicle speed were proposed.

Specifically, the findings highlight the critical roles of road geometry, driver factors, and vehicle speed. As shown by the following points.

1. Sag-curves and crest-curves had higher average lane departure frequencies compared to downslope- and upslope-curves. This may be because the crest-curves would make the horizontal curve look sharper, while the sag-curves would make the horizontal curve look flatter^28^. The ratio of horizontal curvature to vertical curvature significantly influences the degree of departure on combined curves, thereby exacerbating this risk^24^. 2) IDCF events showed greater departure (0.83 m > 0.41 m) and longer departure duration distance (70.07 m > 58.60 m) than ADCF events. 3) The years of driving, daily driving distance, driving experience, and departure duration distance had significantly influence on departure severity. Also, driver characteristics and lane departure duration distance had an interaction effect on departure severity. 4) Speed and road geometry design could jointly influence the degree of lane departure, e.g., speed significantly affects lane departure behaviour on downslope-curves and upslope-curves in the IDCF scenario. 5) The lane departure duration distance threshold (35.00–110.00 m), and speed threshold (86.77–108.63 km/h) were proposed for the lane departure process.

The research can be used to optimize current assisted driving systems (e.g. LDW, speed control). The driver-specific research suggested that driver training and road safety education should be designed to improve the drivers’ ability to cope in complex road environments. Targeted training should also be designed especially for drivers with less driving experience. The study also provided practical guidance for traffic safety management and road design. For example, in sag-curve or crest-curve road sections, the visual guidance design was optimized based on departure duration distance thresholds (e.g., graduated visual guidelines). The combination of comprehensive traffic safety education and road geometric design optimization may be more effective in reducing the risk of lane departure and the probability of traffic accidents.

The limitations of the study are: 1) This article mainly focuses on lane departure behavior. It does not take into account individual differences among drivers when they deviate, such as driver types (including typical, sluggish, and aggressive types. 2) Tracking the behavioral changes of drivers after training to assess the dynamic adjustment mechanism of departure duration distance thresholds. 3) Quantifying the neural mechanism of the association between risk perception and departure duration distance thresholds in combination with eye-tracking or physiological sensor data.

## Supplementary Information

Below is the link to the electronic supplementary material.

Supplementary Material 1

## Data Availability

The data are not publicly available due to a confidentiality agreement signed during the experiment.
