# Supplementary material for: Lane departure on combined curves: driver heterogeneity, centrifugal risk, and crash prevention
Source: Sci Rep. 2026 Feb 12;16:8586. doi: 10.1038/s41598-026-37251-1 (PMC12976100; doi:10.1038/s41598-026-37251-1)
Supplement: Supplementary file 1 — Supplementary Material 1 [file 41598_2026_37251_MOESM1_ESM.pdf]

Highlights

1. Lane departure events occur more frequently on sag-curves and crest-curves than on downslope- and upslope-curves.
2. IDCF (In the Direction of Centrifugal Force) events exhibit greater lateral departure and longer duration than ADCF (Against the Direction of Centrifugal Force) events.
3. Driver experience, crash history, license type, and departure duration distance significantly affect lane departure severity.
4. Speed and road geometry interactively influence the severity of lane departure behaviour.
5. Thresholds for departure duration distance (IDCF) and speed ranges (ADCF) are proposed to support crash prevention strategies.
